# Local control of T cell fate in lymph nodes safely and durably reverses myelin-driven autoimmunity

**DOI:** 10.1073/pnas.2409563122

**Published:** 2025-11-03

**Authors:** Senta M. Kapnick, Emily A. Gosselin, Shannon J. Tsai, Robert S. Oakes, Zahra A. Habibabady, Marian A. Ackun-Farmmer, Sean T. Carey, Shrey A. Shah, Ruochen Shen, Eugene Froimchuk, Haleigh B. Eppler, Christopher J. Bridgeman, Alexis A. Yanes, Ryan A. McIlvaine, Maeesha Noshin, Lisa H. Tostanoski, Sheneil K. Black, Xiangbin Zeng, Agnes Azimzadeh, Richard N. Pierson, Jonathan S. Bromberg, Christopher M. Jewell

**Affiliations:** ^a^Robert E. Fischell Institute for Biomedical Devices, University of Maryland, College Park, MD 20742; ^b^Department of Veterans Affairs, Veterans Affairs Maryland Health Care System, Baltimore, MD 21201; ^c^Fischell Department of Bioengineering, University of Maryland, College Park, MD 20742; ^d^Department of Surgery, Center for Transplantation Sciences, Massachusetts General Hospital, Boston, MA 02114; ^e^Department of Surgery, University of Maryland School of Medicine, Baltimore, MD 21201; ^f^Center for Vascular and Inflammatory Diseases, University of Maryland School of Medicine, Baltimore, MD 21201; ^g^Department of Microbiology and Immunology, University of Maryland School of Medicine, Baltimore, MD 21201; ^h^Marlene and Stewart Greenebaum Comprehensive Cancer Center, University of Maryland, Baltimore, MD 21201

**Keywords:** tolerance, immunotherapy, biomaterials, immune engineering, nanotechnology

## Abstract

Development of tolerogenic, antigen-specific immunotherapies could overcome limitations of existing treatments for inflammatory autoimmune diseases by achieving potent, durable remission without impacting healthy immune surveillance. Here, we deliver diffusion-limited polymer depots to lymph nodes to locally guide T cell fates for the treatment of multiple sclerosis (MS), an autoimmune disease that occurs when the immune system mistakenly attacks myelin. In preclinical MS models, depots loaded with myelin self-antigen and tolerizing cues mediate localized retention of activated CD4 T cells, promote myelin-specific regulatory T cells, and reshape inflammation in the central nervous system (CNS) to eliminate lesions. Selective disease reversal is achieved with a single treatment that induces long-lasting remission without hindering healthy responses to vaccine challenge with foreign antigen. Furthermore, depots offer favorable chemistry and manufacturing control features and are well tolerated in non-human primates. This work supports a clinically feasible concept for inducing safe, effective, antigen-specific tolerance without systemic or repeated dosing.

## Significance

Treatments for multiple sclerosis (MS)—an inflammatory autoimmune disease—rely on systemic dosing, are noncurative, and leave patients vulnerable to infection. Here, we advance a lymph node targeting strategy that exploits local dosing and retention to guide T cell fates. We achieve durable efficacy with a single treatment in multiple preclinical models and show favorable profiles for safety in non-human primates and for chemistry and manufacturing controls (CMC). Mechanistic findings reveal how antigen-specific, tolerogenic T cells are reprogrammed, demonstrating a paradigm to overcome hurdles facing systemic tolerance induction strategies (e.g., antigen-coupled myeloid cells, mRNA vaccines, genetic engineering). Together, these results progress the technology toward human translation as a potential therapy to treat autoimmune disease with antigen-specific selectivity.

Multiple sclerosis (MS) is an autoimmune disorder impacting 2.8 million individuals worldwide (1). MS occurs when proinflammatory lymphocytes mistakenly target myelin, the protective sheath insulating axons of the central nervous system (CNS) (2). Demyelination results in severe disabilities including pain, motor deficits, and cognitive deterioration (1). Although there are over 20 approved disease-modifying therapies (DMTs), none are curative and many leave patients vulnerable to opportunistic infections or interfere with vaccination (3). Thus, next-generation MS therapies that restore peripheral tolerance to myelin without impacting healthy immunity must be both potent and selective.

Many experimental approaches to antigen-specific tolerance have thus far centered on ex vivo generation of regulatory T cells (T_regs_) for suppression of autoreactive cells (4). However, strategies including conditioning (5, 6) and genetic engineering (7–10) are limited by major histocompatibility complex (MHC) restriction, potential concerns with lentiviral-based technologies, high cost, and challenges associated with low T_reg_ frequency in peripheral blood (11). These obstacles have motivated exploration of immunotherapies that instead generate antigen-specific T_regs_ in vivo (12–14). We posited that directly programming the LN niche could further transform the MS treatment landscape by local guidance of myelin-specific T cells in lymphoid tissue without systemic drug infusion or cell therapies. We have previously reported in proof-of-principle studies intra-LN (*i.ln*.) injection concentrates and retains immunomodulatory cues when coupled with diffusion-limited microparticles (MPs) designed to be too large to drain from treated tissue (15). Underscoring this strategy, efficacy does not occur after injection of depots via other routes, or following local LN injection of soluble cargo (15). Further understanding the role of myelin-specific T cell differentiation and function in such approaches could enable clinical success in related human therapies to selectively treat MS.

Here, we address this gap by elucidating key mechanisms underlying our strategic approach. We demonstrate accumulation of clonally distinct T cells in individual LNs containing MP-derived regulatory cues. Diverse antigens drive de novo polarization of T cells to stable, antigen-specific T_regs_ that are highly suppressive. Transcriptional analysis reveals treatment alters signaling pathways that directly control trafficking of T cells. These effects correlate with elimination of spinal cord lesions and disruptive, long-lasting efficacy in multiple autoimmune models following a single treatment. Furthermore, we show that this vaccine-like therapy does not interfere with healthy T and B cell responses during simultaneous challenge with foreign antigen or impede peripheral tolerance. Finally, LN depots offer favorable safety outcomes in non-human primates (NHPs) and an attractive profile for chemistry and manufacturing controls (CMC). This work supports a clinically feasible concept for inducing safe, potent, and myelin-specific tolerance without systemic or repeated dosing.

## Results

### *I.ln.* MPs Create Local Niches that Drive Antigen-Specific T Cells and Promote T_regs_.

**Fig. 1.**
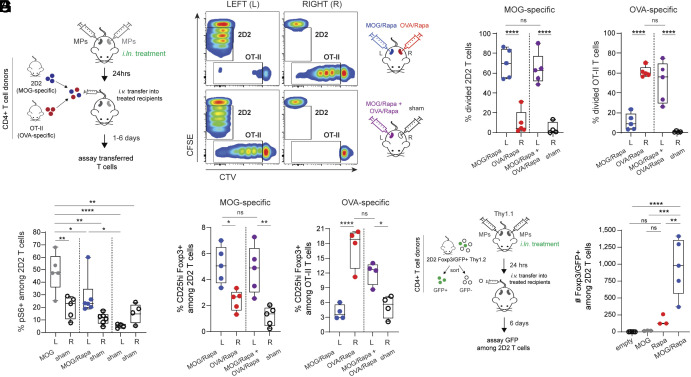
MPs drive local programming of T_regs_ in LNs in an antigen-dependent manner. (*A*) Schematic illustrating design for transfer studies (panels *B*–*E*). (*B*) Representative plots showing proliferation among transferred cells in mice differentially treated in left (L) and right (R) LNs. (*C*) Frequency of divided cells in LNs among MOG-specific 2D2 (*Left* panel) or OVA-specific OT-II T cells (*Right* panel), three days after transfer. Single data points represent individual LNs. (*D*) Frequency of pS6+ 2D2 T cells in left (L) and right (R) LNs, 24h after transfer. (*E*) Quantification of T_regs_ in LNs among MOG-specific 2D2 (*Left* panel) or OVA-specific OT-II T cells (*Right* panel), six days after transfer. (*F*) Schematic illustrating design of transfer studies (panel *G*). (*G*) Number of Foxp3/GFP+ 2D2 T cells among T_reg_-depleted donor cells, six days after transfer. Error bars represent mean ± SD. Dotted vertical lines separate *i.ln.* treatment cohorts for individual mice. ns = not significant, *P < 0.05, **P < 0.01, ***P < 0.001, ****P < 0.0001 by one-way ANOVA Tukey−Kramer multiple comparisons test.

We hypothesized *i.ln.* MPs would generate T cell niches in an antigen-specific manner and that inclusion of the regulatory cue—rapamycin (Rapa)—would promote T_regs_ among these populations. To test this, we treated mice with MPs coencapsulating Rapa and peptide antigen derived either from myelin oligodendrocyte protein (MOG) or a model protein, ovalbumin (OVA) via direct injection into inguinal LNs. Twenty-four hours after treatment, all cohorts received intravenous (*i.v.*) infusions of a 1:1 mixture of 2D2 (MOG-specific) and OT-II (OVA-specific) transgenic donor CD4 T cells (Fig. 1*A*). Dyes with distinct spectral profiles were used to track donor cell frequencies (*SI Appendix*, Fig. S1 *A* and *B*) and proliferated cells in treated and untreated lymph nodes (Fig. 1*B* and *SI Appendix*, Fig. S1 *C* and *D*). Strikingly, 2D2 (MOG-specific) cells proliferated in LNs treated with MOG/Rapa MPs three days after transfer, while 2D2 cells isolated from OVA/Rapa-treated LNs in the same mice exhibited minimal proliferation (Fig. 1 *B* and *C*). Likewise, OVA-specific T cells proliferated in LNs treated with OVA/Rapa MPs but not MOG/Rapa MPs. Excitingly, when we administered MOG/Rapa MPs and OVA/Rapa MPs to the same LN, both populations of transgenic T cells divided, but proliferating cells were absent in the sham-injected contralateral LNs (Fig. 1 *B* and *C*). Likewise, divided cells were not recovered in distal, untreated cervical LNs three days after adoptive transfer (*SI Appendix*, Fig. S1*B*). Thus, *i.ln*. MPs create local niches that control clonally distinct T cells in response to diverse antigens.

Given this antigen-specific, highly localized proliferation, we tested whether Rapa also locally alters T cell signaling by quantifying phosphorylation of ribosomal protein S6 (pS6)—a downstream target of the mechanistic target of rapamycin (mTOR) complex 1 (mTORc1) that is inhibited by rapamycin (16). Cohorts were treated in left LNs with MOG or MOG/Rapa MPs and right LNs with sham injections, or sham injections in both LNs, followed by adoptive transfer of 2D2 T cells (*SI Appendix*, Fig. S2*A*). Twenty-four hours later, pS6+ donor cells recovered from LNs treated with MOG/Rapa MPs decreased relative to MOG MPs (Fig. 1*D* and *SI Appendix*, Fig. S2*B*) included as a baseline control. These data indicate Rapa in MPs inhibits mTORc1 activity in antigen-specific cells in treated LNs.

We next investigated how localized, *i.ln.* MP treatment precisely impacts antigen-specific T_regs_. First, mice were treated with MOG/Rapa MPs (left LN) and OVA/Rapa MPs (right LN), or both MP forms in a single (left) LN and a contralateral sham injection (right LN). Six days after adoptive transfer, we observed an increase in the frequency (Fig. 1*E*) and number (*SI Appendix*, Fig. S3*A*) of T_regs_ among 2D2 cells in MOG/Rapa-treated LNs, compared with 2D2 cells in OVA/Rapa-treated contralateral LNs within the same animal. Likewise, T_regs_ among OT-II T cells were higher in OVA/Rapa-treated LNs compared with MOG/Rapa MPs. T_regs_ among 2D2 and OT-II cells were increased in LNs treated with both MOG/Rapa and OVA/Rapa MP formulations (Fig. 1*E* and *SI Appendix*, Fig. S3*A*). No changes were observed in CD4+ T cell frequencies among non-antigen-specific, recipient cells in MOG/Rapa-treated mice (*SI Appendix*, Fig. S3*B*), and no differences were observed in T_reg_ frequencies in distal, untreated cervical LNs following adoptive transfer (*SI Appendix*, Fig. S3*C*), supporting the specificity and localized nature of the programming paradigm. Importantly, the frequency of recovered, antigen-specific donor T_regs_ was greater than the proportion of T_regs_ transferred into recipients (*SI Appendix*, Fig. S3*D*), indicating MPs simultaneously support distinct, antigen-specific T_reg_ populations within a single LN.

Induced T_regs_ have been shown to re-establish tolerance when T_regs_ are absent or ineffective (17, 18). To assess whether MOG/Rapa MPs generate induced T_regs_ from naive CD4 T cells in the periphery, we sorted GFP-, naive CD4+ T cells from 2D2 Foxp3/GFP mice (*SI Appendix*, Fig. S3*D*), and adoptively transferred T_reg_-depleted cells into MP-treated, congenic recipients (Fig. 1*F*). While Rapa MPs increased the frequency of T_regs_ among transferred cells (*SI Appendix*, Fig. S3*E*), only MOG/Rapa MP-treated recipients exhibited a concomitant increase in numbers of GFP+ donor cells (Fig. 1*G*). These data highlight the need for codelivery of antigen and Rapa and show *i.ln.* treatment promotes T_regs_ in treated tissues.

### Antigen-Specific T_regs_ are Suppressive, Long-Lasting, and Provide Durable Protection Against Autoimmunity.

**Fig. 2.**
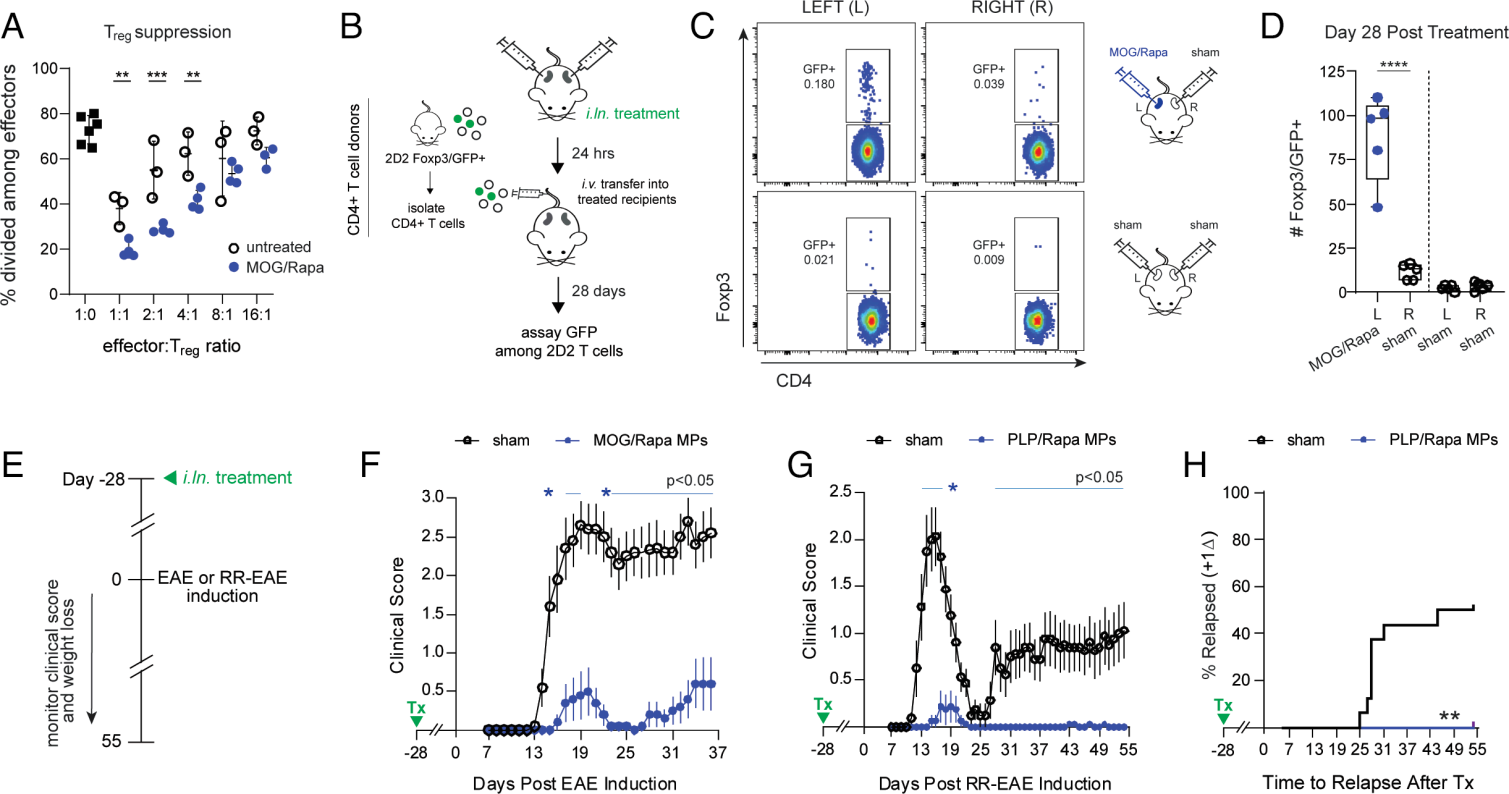
Antigen-specific T_regs_ are suppressive, long-lasting, and provide durable protection against autoimmunity. (*A*) Frequency of divided CD4 T cells at indicated ratios in suppression assays. (*B*) Schematic illustrating design for T_reg_ persistence studies (panels *C* and *D*). (*C*) Representative plots for antigen-specific T_regs_ recovered from *i.ln.* treatment cohorts. (*D*) Quantification of Foxp3/GFP+ T cells recovered from LNs. (*E*) Timeline for durability studies (panels *F*–*H*). Clinical score in EAE (*F*) or RR-EAE mice (*G*) following day −28 *i.ln.* treatment (Tx). (*H*) Relapse incidence in RR-EAE mice. Error bars represent mean ± SD. Dotted vertical lines separate *i.ln.* treatment cohorts for individual mice. Clinical scores represent mean ± SEM. sham n = 16, MOG/Rapa MPs n = 8, PLP/Rapa MPs, n = 15. ns = not significant, *P < 0.05, **P < 0.01, ****P < 0.0001 by Steel–Dwass test (clinical scores), two-way ANOVA with Tukey–Kramer post hoc test (weight loss), Wilcoxon test for Kaplan–Meier analyses, or one-way ANOVA Tukey−Kramer multiple comparisons test.

To investigate whether T_regs_ are functionally suppressive, we sorted GFP+ CD4 T cells from LNs of untreated or MOG/Rapa MP-treated Foxp3/GFP mice for coculture in a suppression assay with naive, effector CD4 T cells stained with proliferative dye (19). T_regs_ from MOG/Rapa-treated LNs exhibited enhanced suppression of effector T cell proliferation compared with T_regs_ isolated from untreated animals (Fig. 2*A* and *SI Appendix*, Fig. S4*A*). Thus, MOG/Rapa MPs increase both the number of antigen-specific T_regs_ and their suppressive capacity on a per cell basis.

One uncertainty faced by T_reg_-based therapeutic strategies to treat autoimmunity is whether T_regs_ are maintained to enable durable tolerance (20, 21). Thus, we examined whether MOG-specific T_regs_ in MP-treated LNs are long-lasting. Mice received a single injection of MOG/Rapa MPs in the left LN only, followed by *i.v.* infusion of CD4 T cells isolated from 2D2 Foxp3/GFP mice (Fig. 2*B*). One month after transfer, GFP+ cells were recovered from MOG/Rapa MP-treated LNs, but were absent or minimal in sham-injected LNs (Fig. 2 *C* and *D* and *SI Appendix*, Fig. S4*B*). This indicates that myelin-specific T_regs_ are locally and stably maintained in treated LNs.

To examine whether long-lived MOG-specific T_regs_ correlate with lasting protection in a preclinical MS model, experimental autoimmune encephalomyelitis (EAE), we treated mice once with *i.ln*. MOG/Rapa MPs 28 d before EAE induction (Fig. 2*E*). Animals were protected from paralysis measured by increases in clinical score indicative of disease severity (Fig. 2*F* and *SI Appendix*, Fig. S4*C*). Likewise, in a model of relapsing-remitting (RR) MS that mimics aspects of cyclic disease observed in MS patients, near-complete protection was observed in mice treated with MPs containing Rapa and proteolipid protein (PLP)—the disease-associated antigen in RR-EAE (Fig. 2*G* and *SI Appendix*, Fig. S4*D*). Efficacy in RR-EAE was durable with no relapse after initial disease onset during a 55-d monitoring period, despite mice receiving only a single treatment 83 d earlier. (Fig. 2*H*). These data indicate peptide/Rapa MPs promote myelin-specific, suppressive T_regs_ that are durable and correlate with long-lasting, vaccine-like protection in progressive and RR MS models.

### A Single MP Treatment Confers Efficacy at Disease Onset, Reverses Paralysis in Established Disease, and Stabilizes Chronic Disease.

**Fig. 3.**
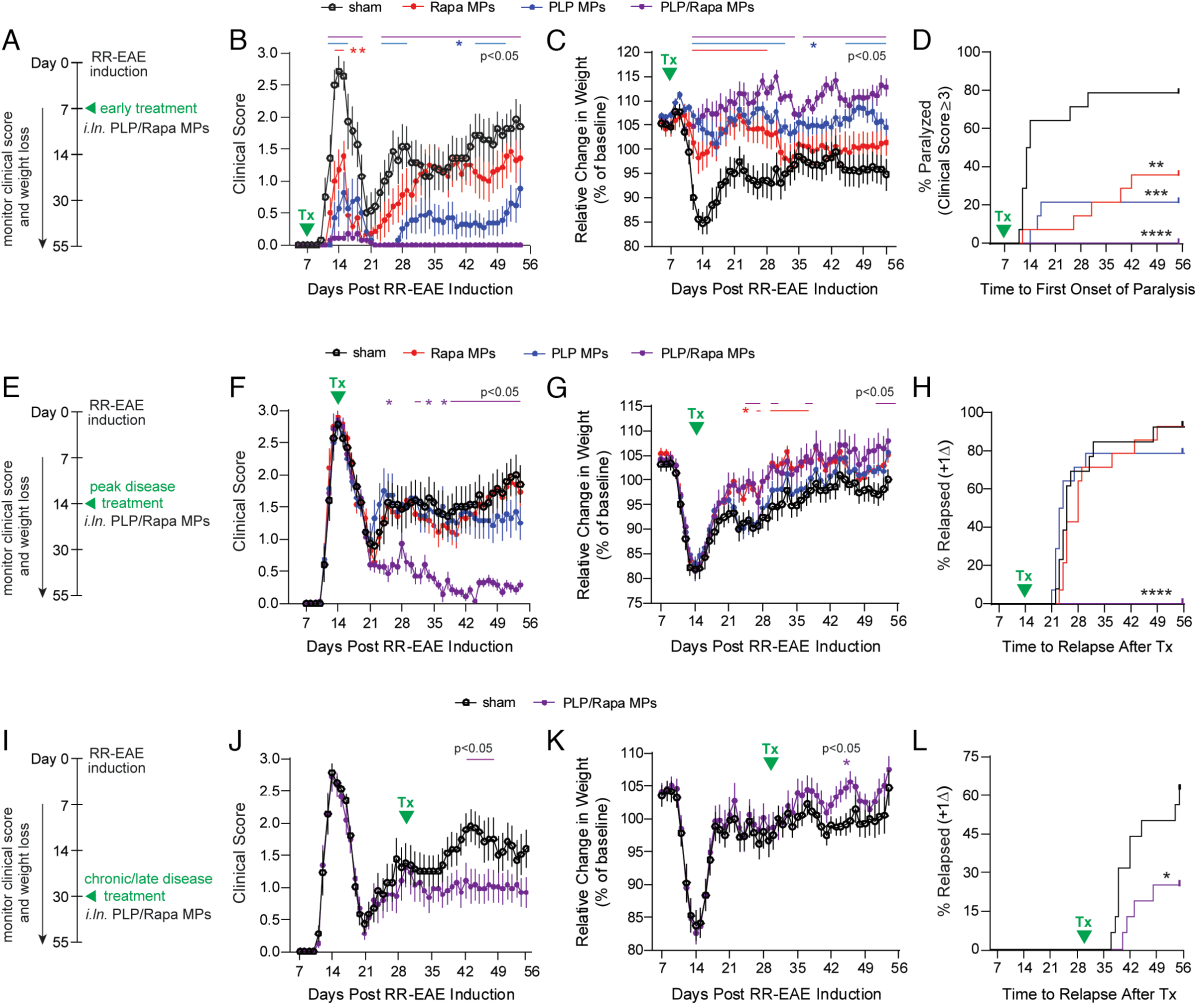
Single-dose *i.ln.* MP treatment provides protection against RR-EAE in both early and chronic therapeutic settings. (*A*) Timeline for early therapeutic MP treatment (panels *B*–*D*). Clinical score (*B*), weight loss (*C*), and paralysis incidence (*D*) in RR-EAE mice treated on day 7. (*E*) Timeline for peak disease MP treatment (panels *F*–*H*). Clinical score (*F*), weight loss (*G*), and relapse incidence (*H*) in RR-EAE mice treated on day 14. (*I*) Timeline for chronic/late disease MP treatment (panels *J*–*L*). Clinical score (*J*), weight loss (*K*), and relapse incidence (*L*) in RR-EAE mice treated on day 30. n=12 to 16 mice/treatment group. Error bars represent mean ± SEM. ns = not significant, *P < 0.05, **P < 0.01, ***P < 0.001, ****P < 0.0001 by Steel–Dwass test (clinical scores), two-way ANOVA with Tukey–Kramer post hoc test (weight loss), or Wilcoxon test for Kaplan–Meier analyses.

To test whether a single *i.ln*. MP treatment provides efficacy across the RR disease course, mice were first treated with PLP/Rapa MPs (supp. Table 1) seven days after RR-EAE induction, before onset of symptoms (Fig. 3*A*). Only MPs coloaded with both PLP and Rapa completely protected against disease (Fig. 3*B* and *SI Appendix*, Fig. S5*A*) and minimized weight loss (Fig. 3*C*). Notably, no paralysis was observed in PLP/Rapa-treated animals only, defined as a clinical score ≥3 (Fig. 3*D*). Then, 79% (11/14) of animals treated with PLP/Rapa MPs were asymptomatic throughout the disease course (*SI Appendix*, Fig. S5*B*). In contrast, 100% of untreated (14/14) or Rapa MP-treated (14/14) animals and 64% (9/14) of PLP MP-treated animals developed symptoms, indicated by scores > 0 (*SI Appendix*, Fig. S5*B*). This result is consistent with studies reporting that peptide only impacts severity of the disease course (22–25). Notably however, all mice in the PLP/Rapa MP, but not PLP or Rapa MP cohorts, fully recovered from onset of initial symptoms and were resistant to relapse throughout the study (Fig. 3*B*).

Next, we determined whether MPs could control established disease using a more challenging scenario: a single MP treatment at peak disease (day 14) (Fig. 3*E*). PLP/Rapa MPs prevented relapse, and excitingly, reversed disease and weight loss to nearly asymptomatic levels (Fig. 3 *F* and *G*). All mice in other treatment cohorts relapsed (Fig. 3*H*) with no significant differences in disease severity between groups (*SI Appendix*, Fig. S5*C*).

Finally, we tested efficacy using the most challenging regimen: a single MP treatment during established chronic/late-stage disease (day 30) (Fig. 3*I*). PLP/Rapa MPs treatment stabilized disease (Fig. 3 *J* and *K*); 69% (11/16) of sham-treated mice exhibited a second relapse, whereas only 25% (4/16) of PLP/Rapa-treated animals relapsed. Relapse in PLP/Rapa-treated mice was also delayed (Fig. 3*L*) and disease less severe (*SI Appendix*, Fig. S5*D*). Together these data show potent single-dose efficacy across onset, established, and chronic disease, demonstrating potential as a therapeutic vaccine for myelin-specific immunotherapy in MS.

### Efficacy Correlates with Elimination of CNS Lesions.

**Fig. 4.**
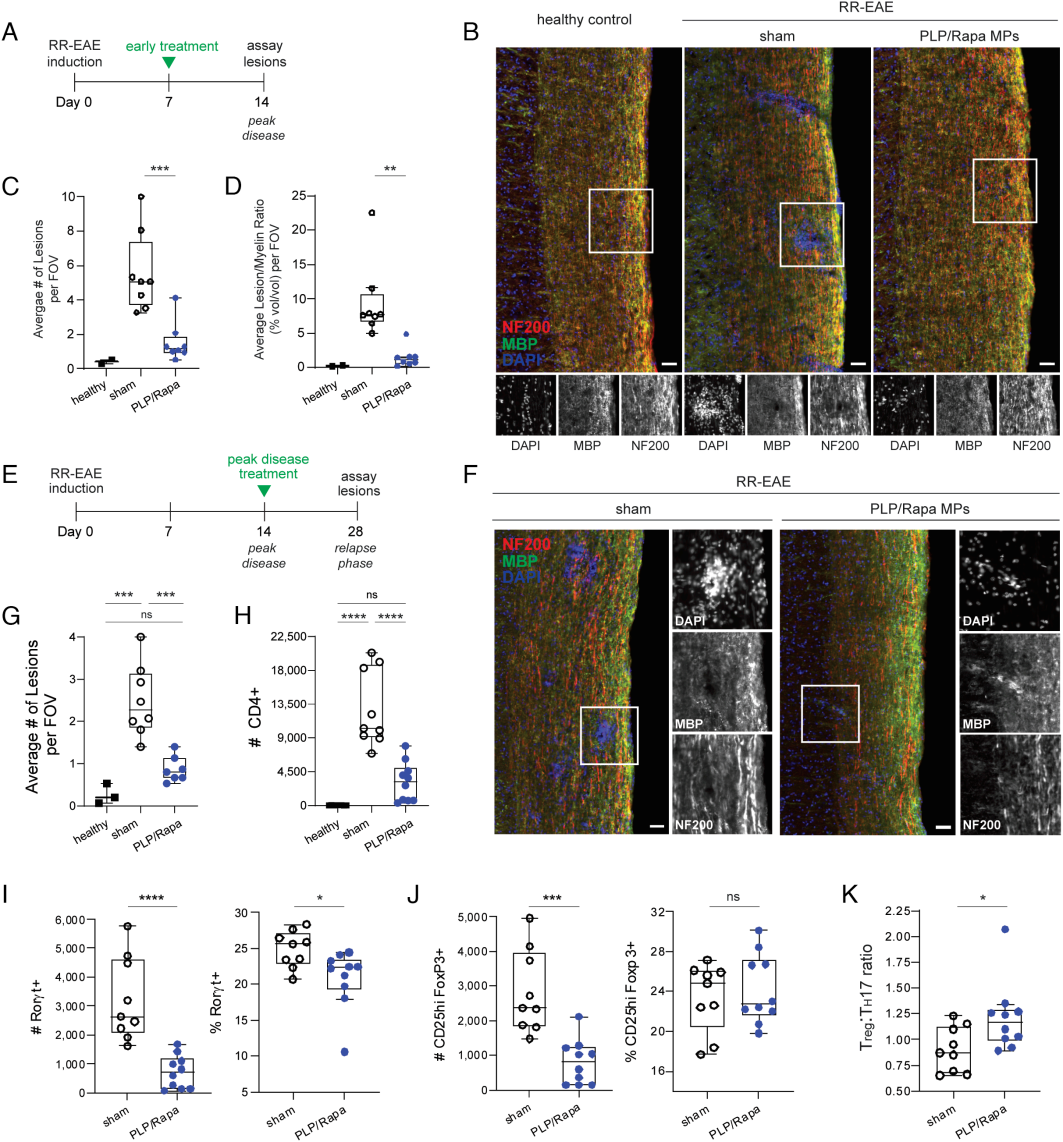
Localized programming in LNs is accompanied by reduced lesions and altered T cell infiltration in the CNS. (*A*) Timeline for analysis of spinal cords following early therapeutic treatment (panels *B*–*D*). (B) Representative images of sectioned spinal cords harvested from healthy (*Left*), sham-injected RR-EAE (*Middle*), or PLP/Rapa MP-treated RR-EAE mice (*Right*). Healthy, n = 2 mice. Sham/RR-EAE, n = 8 mice, PLP/Rapa MP/RR-EAE, n = 8. Average number of lesions (*C*) and lesion/myelin ratio per FOV per mouse (*D*). (*E*) Timeline for analysis of spinal cords following peak disease treatment (panels *F*–*K*). (*F*) Representative images of sectioned spinal cords harvested from sham-injected RR-EAE (*Left*) or PLP/Rapa MP-treated RR-EAE mice (*Right*). Sham/RR-EAE, n = 8 mice, PLP/Rapa MP/RR-EAE, n = 7. (*G*) Average number of lesions per FOV per mouse. (*H*) Quantification of CD4 T cells in spinal cords. (*I*) Quantification of Rorγt+ CD4 T cells (T_H_17s) in spinal cords, number (*Left*) or frequency (*Right*). (*J*) Quantification of CD25hi Foxp3+ CD4 T cells (T_regs_) in spinal cords, number (*Left*) or frequency (*Right*). (*K*) Ratio T_reg_:T_H_17 in spinal cords. Sham, n = 9. PLP/Rapa, n = 10. Boxes in images denote insets. (Scale bars, 50 µm.) Error bars represent mean ± SD. ns = not significant, *P < 0.05, **P < 0.01, ***P < 0.001, ****P < 0.0001 by Student’s *t* test or one-way ANOVA Tukey−Kramer multiple comparisons test.

To connect efficacy to changes in CNS lesions—the root cause of paralysis in patients—spinal cords were harvested and examined via immunofluorescence microscopy. Following an early therapeutic regimen (day 7) (Fig. 4*A* and *SI Appendix*, Fig. S6*A*), PLP/Rapa MP-treated mice exhibited fewer lesions (Fig. 4 *B* and *C*) and lower lesion-to-myelin ratio relative to sham (Fig. 4*D*). Next, we treated mice at the peak of RR-EAE (day 14) and measured lesions at day 28 (relapse phase) (Fig. 4*E* and *SI Appendix*, Fig. S6*B*). During this more challenging regimen, treated animals again exhibited significant reductions in lesions (Fig. 4 *F* and *G*) and the lesion/myelin ratio (*SI Appendix*, Fig. S6*C*). Remarkably, lesion levels in mice treated at peak disease was statistically undistinguishable to healthy mice never induced with RR-EAE.

### *I.ln.* MP Treatment Alters the Inflammatory-To-Regulatory T Cell Balance in the CNS.

Lesion formation is partially mediated by infiltrating autoreactive inflammatory T cells (e.g., T_H_17) (26). Thus, we hypothesized local reprogramming of LNs alters T cell populations in the CNS. A single dose of *i.ln.* PLP/Rapa MPs decreased the number of CD45hi cells (*SI Appendix*, Fig. S7 *A*–*C*), a marker of infiltrating leukocytes, and the number of CD4 T cells in spinal cords when administered as an early intervention (*SI Appendix*, Fig. S7*D*) or during peak disease (Fig. 4*H* and *SI Appendix*, Fig. S7*E*). Among CD4 T cells, peak disease treatment (day 14) reduced the frequency and number of T_H_17s in spinal cords during relapse (Fig. 4*I* and *SI Appendix*, Fig. S7*F*). While we observed a decrease in the total number of T_regs_ in treated cohorts—consistent with the overall reduction in CD4 T cell infiltrates in treated mice—the T_reg_ frequency was unchanged in mice that received PLP/Rapa MPs (Fig. 4*J* and *SI Appendix*, Fig. S7*F*). These data suggest that among the CD4 T cells that traffic to spinal cords in treated mice, albeit less, a larger proportion are regulatory in phenotype—indicated by an increase in a T_reg_:T_H_17 ratio that takes into account the overall reduction in cell infiltrates (Fig. 4*K*). Together, these data reveal that antigen-specific reprogramming in LNs alters T cell populations that reshape inflammation and eliminate lesions in distal CNS tissue sites relevant for MS, without systemic or repeated dosing.

### CD4 T Cell Trafficking Is Altered by Rapa-Mediated Transcriptional Regulation in Treated LNs.

**Fig. 5.**
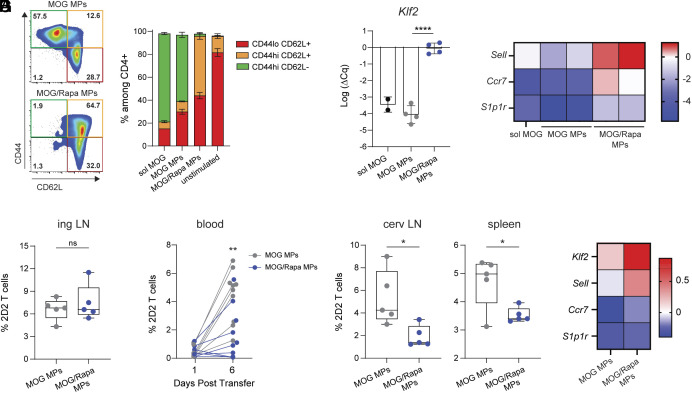
Trafficking of CD4 T cells is altered by Rapa-mediated transcriptional regulation in treated LNs. (*A*) Flow cytometric analyses of CD44 and CD62L surface marker expression, unstimulated or three days after start of DC:T cell in vitro cocultures. Quantification of (*B*) *Klf2* mRNA and (*C*) gene expression analysis of 2D2 T cells from in vitro DC:T cocultures, expressed as relative to untreated. (*D*) Frequency of 2D2 T cells in treated, inguinal LNs six days post transfer into MP-treated recipients. (*E*) Frequency of 2D2 T cells in the blood. Connected points represent paired data from individual mice over time. (*F*) Frequency of 2D2 T cells in untreated tissues, six days post transfer; cervical LN (*Left* panel) and spleen (*Right* panel). (*G*) Gene expression analysis of sorted CD4 T cells pooled from inguinal LNs, expressed as relative to untreated. n = 4 to 5/treatment. Error bars represent mean ± SD, Tukey. ns = not significant, *P < 0.05, **P < 0.01, ****P < 0.0001 by Student’s *t* test or one-way ANOVA Tukey−Kramer multiple comparisons test.

Given the overall reduction in the number of infiltrating CD4 T cells recovered from the CNS of treated mice (Fig. 4*H*), we next investigated whether *i.ln.* treatment impacts trafficking of antigen-specific cells. This hypothesis is motivated by work showing that mTORC1 signaling, which is inhibited by rapamycin, controls expression of homing receptors in activated T cells (27). To test this, we used a primary in vitro coculture system of splenic dendritic cells (DCs) and 2D2 T cells that allows examination of receptor expression in a closed environment. While MOG induced downregulation of CD62L in CD4 T cells, T cells pretreated with MOG/Rapa MPs maintained CD62L expression (Fig. 5*A* and *SI Appendix*, Fig. S8*A*), a LN homing molecule that is lost following T cell receptor engagement. Notably, CD44 expression was similar among treatment groups (Fig. 5*A* and *SI Appendix*, Fig. S8*B*). These data suggest that Rapa in MPs could alter trafficking without impacting activation.

CD62L expression is regulated by the transcription factor, Kruppel-like factor 2 (KLF2), that is suppressed by mTORC1 signaling (27, 28). To test whether MPs exert transcriptional control over KLF2, we performed real-time qPCR (RT-qPCR) analysis on CD4 T cells. Treatment with MOG/Rapa inhibited downregulation of *Klf2* compared with antigen-only controls (Fig. 5*B*). Gene expression profiling further revealed altered expression of KLF2 targets: *Ccr7*, and *S1p1r* that control egress of T cells from LNs (Fig. 5*C*) (29). These data indicate MOG/Rapa MPs transcriptionally regulate *Klf2* and associated homing molecules in antigen-specific CD4 T cells.

To test whether *i.ln.* MPs alter trafficking of activated CD4 T cells in vivo, 2D2 T cells were adoptively transferred into congenic recipients *i.ln.*-treated with either MOG or MOG/Rapa MPs. While the frequency (Fig. 5*D*) and number (*SI Appendix*, Fig. S8*C*) of antigen-specific T cells in treated LNs were similar among groups six days after transfer, we measured significant differences in blood (Fig. 5*E* and *SI Appendix*, Fig. S8*D*) and untreated tissues (i.e., cervical LNs and spleen) (Fig. 5*F*). Gene expression profiling performed on CD4 T cells isolated from treated LNs revealed Rapa in MPs limited *Klf2* and *Sell* downregulation. Correspondingly, reduced downregulation of *Ccr7* expression and to a lesser extent, *S1p1r*, were observed (Fig. 5*G*). Although MOG/Rapa MPs limited proliferation of T cells relative to antigen-only *i.ln.* treatment on day 3 (*SI Appendix*, Fig. S8*E*), when dye could be reliably used to track division, these data indicate that differences in circulation are not solely due to changes in proliferative capacity. Thus, peptide/Rapa MPs exert local control over transcriptional programs that alter trafficking of activated T cells in treated tissues and potentially limit peripheral damage by autoreactive cells.

### Healthy, Systemic Responses to Vaccination are Intact Following *i.ln.* MP Treatment.

**Fig. 6.**
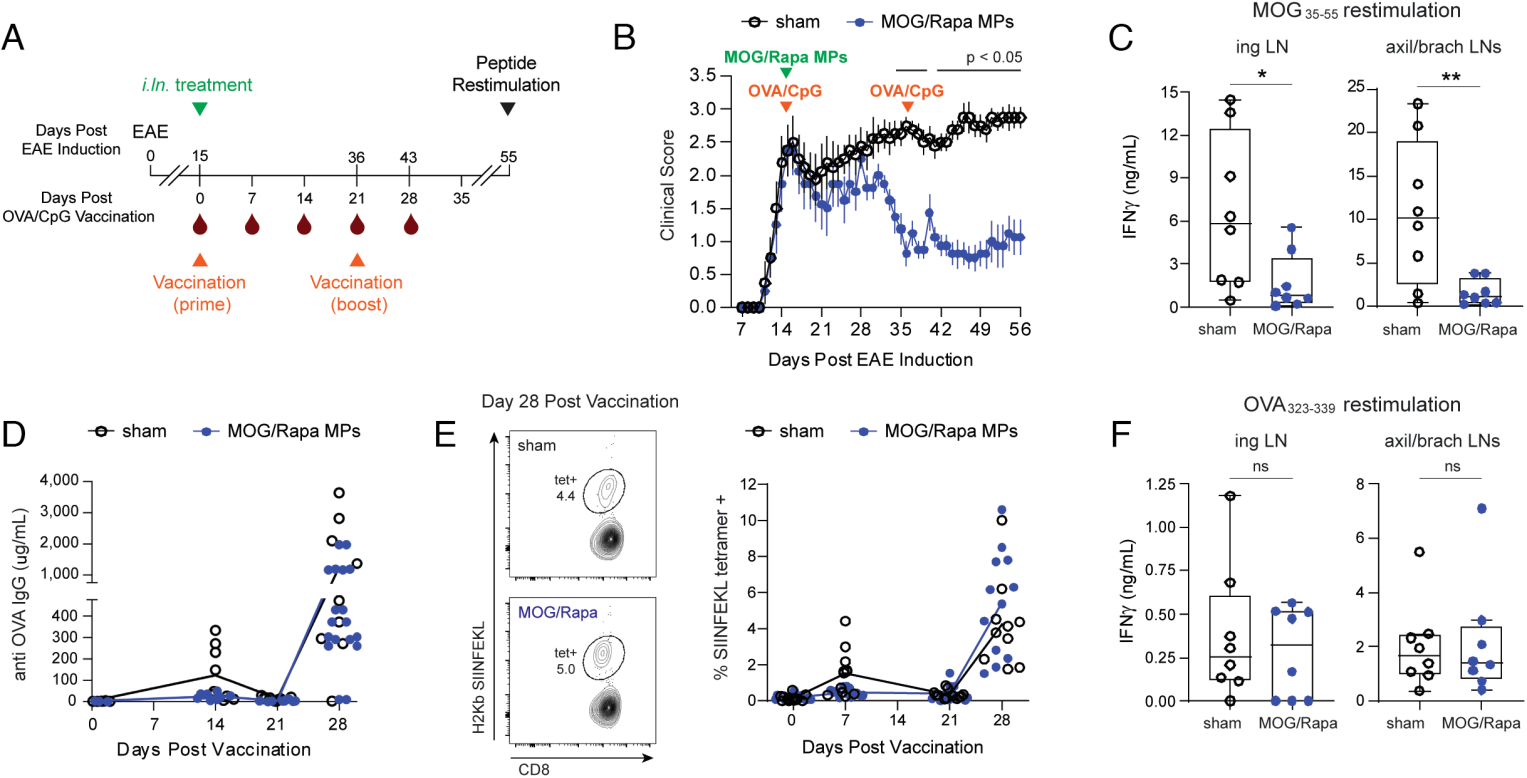
*I.ln.* MP treatment does not impact healthy immune responses to vaccination. (*A*) Timeline for treatment and analysis of immune responses in treated, vaccinated, EAE mice. (*B*) Clinical score. (*C*) IFNγ secretion by cells recovered from inguinal (*Left*) or axillary/brachial (*Right*) LNs following MOG_35-55_ restimulation. (*D*) Anti OVA IgG in serum over time. (*E*) Representative gating strategy (*Left*) and quantification (*Right*) of the CD8 SIINFEKL tetramer+ cells over time. (*F*) IFNγ secretion by cells recovered from inguinal (*Left*) or axillary/brachial (*Right*) LNs following OVA_323-339_ restimulation. Sham, n = 9. MOG/Rapa MPs, n=10. Clinical scores represent mean ± SEM. Error bars represent mean ± SD. ns = not significant, *P < 0.05, **P < 0.01, ***P < 0.001, ****P < 0.0001 by Steel–Dwass test (clinical score), two-way ANOVA with Tukey–Kramer post hoc or Student’s *t* test.

Antigen-specific immunotherapies that preserve healthy immunity by limiting off-target effects would be disruptive for the MS therapeutics landscape. To test whether *i.ln.* MPs leave proinflammatory responses to foreign antigen intact, mice with EAE were simultaneously treated with *i.ln.* MPs and subcutaneously immunized using a vaccine composed of ovalbumin (OVA) protein admixed with CpG adjuvant (30) (Fig. 6*A*). The efficacy of MP treatment was unaffected by vaccination (Fig. 6*B* and *SI Appendix*, Fig. S9*A*) and was accompanied by a reduction in antigen-specific recall responses during MOG peptide restimulation of cells from treated LNs that is suggestive of tolerance (Fig. 6*C*). Importantly, MP treatment did not inhibit OVA-specific vaccine responses, measured by anti OVA IgG antibody production (Fig. 6*D* and *SI Appendix*, Fig. S9*B*) and tetramer+ CD8 T cell frequency (Fig. 6*E* and *SI Appendix*, Fig. S9*C*), or suppress IFNγ production in response to OVA peptide restimulation (Fig. 6*F* and *SI Appendix*, Fig. S9*D*). These data demonstrate local conditioning of LNs with MOG/Rapa MPs drives systemic, myelin-specific tolerance without hindering simultaneous protective, antigen-specific responses to vaccine challenge. Technologies offering this selectivity would address important risks faced by patients receiving existing autoimmune therapies.

### Lyophilized, Stored MPs Retain Molecular and Immunological Fidelity.

CMC considerations are vital to ensure therapeutics are well-defined and intact during synthesis, storage, and administration. We lyophilized and stored MOG/Rapa MPs for 90 d before reconstitution (31), then assessed the biophysical and biochemical properties of MP cargo. Comparison of functional groups using NMR (*SI Appendix*, Fig. S10 *A*–*C*) and mass spectrometry (*SI Appendix*, Fig. S11 *A* and *B*) revealed molecular equivalency between free, soluble MOG and Rapa standards and their counterparts recovered from MPs after lyophilization, storage, and reconstitution (*SI Appendix*, Table S2). Likewise, equivalent efficacy in EAE was observed between reconstituted and freshly prepared MPs using early (*SI Appendix*, Fig. S12*A*) or peak disease (*SI Appendix*, Fig. S12*B*) interventions. These studies support favorable CMC characteristics for patient translation.

### *I.ln.* Delivery of MPs Exhibits a Favorable Safety Profile in NHPs.

**Fig. 7.**
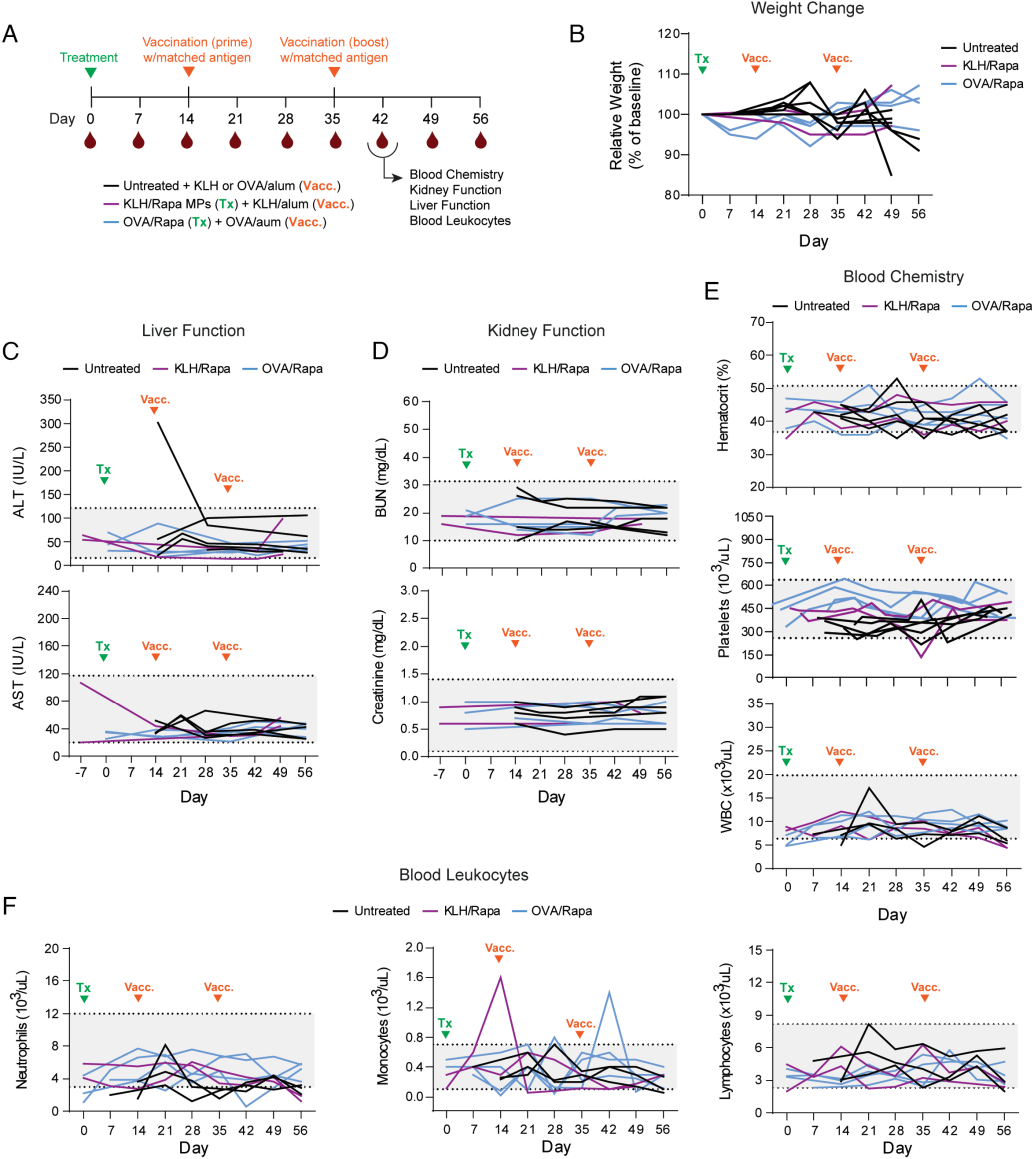
*I.ln.* delivery of MPs exhibits a favorable safety profile in non-human primates. (*A*) Timeline for cynomolgus macaque safety study. (*B*) Relative change in weight over time. (*C*) Liver function parameters—alanine transaminase (ALT) (*Top*) and aspartate aminotransferase (AST) (*Bottom*). (*D*) Kidney function parameters—blood urea nitrogen (BUN) (*Top*) and creatinine (*Bottom*). (*E*) Total percent hematocrit (*Top*), platelets (*Middle*), and white blood cells (WBC) (*Bottom*). (*F*) Total circulating neutrophils (*Left*), monocytes (*Middle*), and lymphocytes (*Right*). Untreated, n = 4 to 6; KLH/Rapa MPs, n = 2; OVA/Rapa MPs, n = 4.

Finally, we tested whether *i.ln.* MPs avoid safety and tolerability concerns associated with systemic therapies [e.g., mild, transient hepatoxicity and nephrotoxicity observed with mTOR inhibitors, attributable to high trough drug levels (32) or hyperinsulinemic hypoglycemia (33, 34)]. NHPs were treated *i.ln.* with MPs coloaded with Rapa and either OVA or keyhole limpet hemocyanin (KLH)—a prototypic T-helper-dependent immunogen commonly used in NHP and human studies (35) (Fig. 7*A* and *SI Appendix*, Table S3). Single-dose treatment did not alter weight (Fig. 7*B*), liver (ALT, AST; Fig. 7*C*), or kidney function (BUN, creatinine; Fig. 7*D*), suggesting that *i.ln.* MP treatment exhibits favorable safety in NHPs. We also tested whether introduction of antigen to LNs causes unwanted proinflammatory responses by challenging MP-treated monkeys intramuscularly with matched antigen in adjuvant (Fig. 7*A*). Hematocrit, platelets, and white blood cell count (WBC) remained within normal ranges (Fig. 7*E*). No gross perturbations indicative of a systemic inflammatory response were detected, as circulating neutrophils, monocytes, lymphocytes (Fig. 7*F*), and lymphocyte subsets (*SI Appendix*, Fig. S13 *A*–*D*) were unchanged. Additionally, intact initial and secondary antibody responses to vaccination were observed (*SI Appendix*, Fig. S13 *E* and *F*).

Interestingly, one animal immunized with KLH/Rapa MPs exhibited an IgM response following treatment but before vaccination (*SI Appendix*, Fig. S13*E*). However, the number of observations is insufficient to assess sensitizing effects. Thus, we examined this possibility of sensitization by assessing B cell responses in mice. To test this, healthy mice were treated *i.ln.* with MPs assembled with Rapa and either OVA_323-339_ peptide or whole OVA protein. MPs did not induce anti OVA IgG, compared with healthy, untreated mice or control mice vaccinated with OVA plus CpG using a prime/boost strategy that produces detectable circulating antibodies (*SI Appendix*, Fig. S13*G*). Collectively, these data suggest *i.ln.* MPs appear safe and unlikely to trigger pathogenic autoimmunity (36).

## Discussion

Our data suggest a model (*SI Appendix*, Fig. S14) where antigen-specific T cells in MP-treated LNs undergo TCR engagement in a localized manner. Rapa creates tolerizing conditions that favor T_reg_ polarization (37–40) and impact trafficking of activated T cells, limiting damage of peripheral tissue. Together, these findings provide insight into the immunological mechanisms contributing to the durable, potent efficacy observed in preclinical models of MS following single-dose treatment. This approach, which relies on LN depots to circumvent systemic dosing that suffers from dose-limited efficacy, off-target effects, and hepatic clearance (41, 42), advances an alternative paradigm to existing immunotherapies that could offer correspondingly disruptive outcomes in patients relative to past clinical trials seeking antigen-specific tolerance through systemic dosing.

Emerging strategies to induction of T_reg_-mediated tolerance for MS treatment (43, 44) have shown promise in Phase 1 trials (23, 45, 46), but less success in Phase II (36, 47) and III (48). *I.ln*. delivery provides unique flexibility for precise, coordinated administration of multiple signals considered classical drivers of tolerance—local display of (self) antigen in the absence of inflammation or in the presence of regulatory milieus (49). Here, we establish these localized signals not only support existing T_regs_ but generate antigen-specific T_regs_ in vivo that exhibit enhanced suppressive function. Persistent T_regs_ are also associated with long-lasting effects in distal, disease-relevant tissues. Future pharmacokinetic (PK) studies examining biodistribution and release rates of tolerogenic signals from MPs in LNs could help inform dose selection and treatment regimen. This information, coupled with potential roles of memory T_reg_ induced by treatment could help inform the durability of therapeutic effects and if retreatment might be beneficial. Notably, induction of tolerogenic memory is an exciting area of growing interest that *i.ln* depots may be able to exploit in pursuit of durable remission (50–52).

In addition, we show that localizing cues made possible by *i.ln.* MPs lead to altered trafficking of activated CD4 T cells that would also mitigate destruction of peripheral tissues. The relevance of this mechanism is evidenced by approved DMTs that inhibit T cell migration in MS [e.g., fingolimod (53, 54)]. Our data suggest that altered mTORc1 signaling by Rapa releases inhibition of KLF2, resulting in maintenance of CD62L and reduced downregulation of CCR7 (27) that promote LN homing and retention. It is not known at this time whether the contribution of CCR7 expression to LN homing in our system outweighs the effects of S1PR1 (55), which promotes T cell egress and is qualitatively less affected. Importantly, the localized retention of T cells mediated by MP depots does not completely abrogate trafficking, and mice exhibit healthy immunity following vaccination, unlike select systemic interventions that globally prevent lymphocyte egress and leave patients immunocompromised.

Our studies demonstrate *i.ln.* treatment is safe in NHPs. A simple surgical procedure was utilized to access inguinal LNs for injections; clinically, this could be replaced by image-guided injection enabling a minimally invasive approach using common technologies employed in biopsies (56). Clinical trials examining the safety and efficacy of intralymphatic immunotherapies (ILIT) for allergen sensitization (57, 58) and treatment of type 1 diabetes (59, 60) have defined target injection volumes (e.g., 0.1 to 0.4mL/LN) and dose ranges (e.g., 12ug GAD-alum/regimen). Injection site decisions will be driven by existing safety findings and accessibility of nodes. Notably, our data have shown that treatment of inguinal LNs in NHPs is safe and confers potent efficacy in mice—the most common set of nodes targeted by existing ILITs. While dose selection and frequency utilized for soluble therapies may not fully align with ILITs using intralymphatic MP depots, these clinical studies using soluble cargo with ILITs lay a solid foundation for future trials testing intra lymphatic MP depots to stop autoimmune disease.

One obstacle associated with existing MS therapeutics is loss of efficacy during severe disease activity, which patients and physicians report as the primary reason for switching DMTs (61). We observed beneficial effects of a single treatment at all intervention times, including during late/chronic disease. These data suggest that MP treatment may also impact remyelination mediated by innate cells that are critical for successful therapeutic outcomes in MS patients with progressive disease (62).

Finally, a persistent hurdle for antigen-specific MS immunotherapy is the identification of definitive target autoantigen(s). However, utilization of encephalitogenic peptides derived from myelin basic protein (MBP) (e.g., MBP_83-99_), proteolipid protein (e.g., PLP_139-154_), and myelin oligodendrocyte glycoprotein (e.g., MOG_1-20_, MOG_35-55_) exhibit immunodominance and have shown promise in MS clinical trials (23, 25, 63, 64). These peptides, and others that continue to surface [e.g., GDP L-fucose synthase (65), TSTA3 (66)], provide opportunities for targeting. Additionally, our data demonstrate that the *i.ln.* MP platform allows for facile exchange of peptides (and proteins) during assembly (e.g., MOG, PLP, OVA, KLH). Incorporation of multiple, diverse antigens could help to address the phenomenon of epitope spread in autoimmunity (29). Related to this, we have previously shown that Rapa MPs assembled with a CD4 peptide relevant for type 1 diabetes offer protection in a CD8-mediated mouse model of disease (49). Furthermore, *i.ln.* delivery of whole protein in MPs also represents a potential strategy for the treatment of autoimmune diseases where B cells and/or autoantibodies mediate disease (67, 68). Thus, diffusion-limited lymph node depots create a potential platform to induce specific and lasting-tolerance in MS or other autoimmune disease.

## Materials and Methods

Materials were assembled as previously described [15, 31]; a detailed description of parameters for particle synthesis, characterization, and biochemical and biophysical analyses (e.g., LC-MS, ^1^H-NMR) can be found in the *Extended Materials and Methods* sections of *SI Appendix*. Cell isolation and culture conditions, including protocols for ELISAs and RT-qPCR, are described in *SI Appendix*. Data from flow cytometric phenotyping and related assays (e.g., T_reg_ suppression, restimulation) were acquired on a BD FACSCelesta or CytoFlex (Beckman Coulter) and analyzed using FlowJo (BD). Cell sorting for adoptive transfers was performed on a BD FACSAria II. For immunofluorescence microscopy, spinal cords were sectioned, stained, and imaged using an Olympus IX83 microscope, as described in *SI Appendix*. All animal work (e.g., vaccination, induction/monitoring for EAE and RR-EAE preclinical MS mouse models, NHP studies) was performed using protocols approved by the Institutional Animal Care and Use Committee (IACUC) overseen by either the University of Maryland at College Park or the University of Maryland School of Medicine. Additional reagent lists and descriptions of statistical analyses are also included in subsections of the *Extended Materials and Methods* of *SI Appendix*.

## Supplementary Material

Appendix 01 (PDF)

## Data Availability

All study data are included in the article and/or *SI Appendix*.

## References

[1] D. S. Reich, C. F. Lucchinetti, P. A. Calabresi, Multiple sclerosis. N. Engl. J. Med. **378**, 169–180 (2018). 29320652 10.1056/NEJMra1401483 PMC6942519

[2] S. Rodriguez Murua, M. F. Farez, F. J. Quintana, The immune response in multiple sclerosis. Annu. Rev. Pathol. **17**, 121–139 (2022). 34606377 10.1146/annurev-pathol-052920-040318

[3] A. Winkelmann, M. Loebermann, E. C. Reisinger, H. P. Hartung, U. K. Zettl, Disease-modifying therapies and infectious risks in multiple sclerosis. Nat. Rev. Neurol. **12**, 217–233 (2016). 26943779 10.1038/nrneurol.2016.21

[4] A. Lutterotti, H. Hayward-Koennecke, M. Sospedra, R. Martin, Antigen-specific immune tolerance in multiple sclerosis-promising approaches and how to bring them to patients. Front Immunol. **12**, 640935. (2021). 33828551 10.3389/fimmu.2021.640935 PMC8019937

[5] J. A. Bluestone , Type 1 diabetes immunotherapy using polyclonal regulatory T cells. Sci. Transl. Med. **7**, 315ra189 (2015). 10.1126/scitranslmed.aad4134 PMC4729454 26606968

[6] K. Chwojnicki , Administration of CD4(+)CD25(high)CD127(−)FoxP3(+) regulatory T cells for relapsing-remitting multiple sclerosis: A phase 1 study. BioDrugs **35**, 47–60 (2021). 33400237 10.1007/s40259-020-00462-7

[7] G. D. Keeler , Gene therapy-induced antigen-specific Tregs inhibit neuro-inflammation and reverse disease in a mouse model of multiple sclerosis. Mol. Ther. **26**, 173–183 (2018). 28943274 10.1016/j.ymthe.2017.09.001 PMC5762980

[8] L. Passerini , CD4(+) T cells from IPEX patients convert into functional and stable regulatory T cells by FOXP3 gene transfer. Sci. Transl. Med. **5**, 215ra174 (2013). 10.1126/scitranslmed.3007320 24337481

[9] Y. C. Kim , Engineered MBP-specific human Tregs ameliorate MOG-induced EAE through IL-2-triggered inhibition of effector T cells. J. Autoimmun. **92**, 77–86 (2018). 29857928 10.1016/j.jaut.2018.05.003 PMC6054915

[10] S. Oh , Precision targeting of autoantigen-specific B cells in muscle-specific tyrosine kinase myasthenia gravis with chimeric autoantibody receptor T cells. Nat. Biotechnol. **41**, 1229–1238 (2023). 36658341 10.1038/s41587-022-01637-z PMC10354218

[11] C. Selck, M. Dominguez-Villar, Antigen-specific regulatory T cell therapy in autoimmune diseases and transplantation. Front Immunol. **12**, 661875 (2021). 34054826 10.3389/fimmu.2021.661875 PMC8160309

[12] R. M. Pearson , Overcoming challenges in treating autoimmunity: Development of tolerogenic immune-modifying nanoparticles. Nanomedicine **18**, 282–291 (2019). 30352312 10.1016/j.nano.2018.10.001 PMC6830541

[13] J. Montano, J. Garnica, P. Santamaria, Immunomodulatory and immunoregulatory nanomedicines for autoimmunity. Semin. Immunol. **56**, 101535 (2021). 34969600 10.1016/j.smim.2021.101535

[14] S. T. Carey, C. Bridgeman, C. M. Jewell, Biomaterial strategies for selective immune tolerance: Advances and gaps. Adv. Sci. (Weinh) **10**, e2205105 (2023). 36638260 10.1002/advs.202205105 PMC10015875

[15] L. H. Tostanoski , Reprogramming the local lymph node microenvironment promotes tolerance that is systemic and antigen specific. Cell Rep. **16**, 2940–2952 (2016). 27626664 10.1016/j.celrep.2016.08.033 PMC5024722

[16] C. Procaccini , An oscillatory switch in mTOR kinase activity sets regulatory T cell responsiveness. Immunity **33**, 929–941 (2010). 21145759 10.1016/j.immuni.2010.11.024 PMC3133602

[17] D. Haribhai , A requisite role for induced regulatory T cells in tolerance based on expanding antigen receptor diversity. Immunity **35**, 109–122 (2011). 21723159 10.1016/j.immuni.2011.03.029 PMC3295638

[18] M. Akamatsu , Conversion of antigen-specific effector/memory T cells into Foxp3-expressing T(reg) cells by inhibition of CDK8/19. Sci. Immunol. **4**, eaaw2707 (2019). 31653719 10.1126/sciimmunol.aaw2707

[19] L. W. Collison, D. A. Vignali, In vitro Treg suppression assays. Methods Mol. Biol. **707**, 21–37 (2011). 21287326 10.1007/978-1-61737-979-6_2 PMC3043080

[20] N. Mikami, R. Kawakami, S. Sakaguchi, New Treg cell-based therapies of autoimmune diseases: Towards antigen-specific immune suppression. Curr. Opin. Immunol. **67**, 36–41 (2020). 32827951 10.1016/j.coi.2020.07.004

[21] S. Sakaguchi, D. A. Vignali, A. Y. Rudensky, R. E. Niec, H. Waldmann, The plasticity and stability of regulatory T cells. Nat. Rev. Immunol. **13**, 461–467 (2013). 23681097 10.1038/nri3464

[22] D. R. Getts , Microparticles bearing encephalitogenic peptides induce T-cell tolerance and ameliorate experimental autoimmune encephalomyelitis. Nat. Biotechnol. **30**, 1217–1224 (2012). 23159881 10.1038/nbt.2434 PMC3589822

[23] A. Lutterotti , Antigen-specific tolerance by autologous myelin peptide-coupled cells: A phase 1 trial in multiple sclerosis. Sci. Transl. Med. **5**, 188ra175 (2013). 10.1126/scitranslmed.3006168 PMC3973034 23740901

[24] R. M. Pearson , Controlled delivery of single or multiple antigens in tolerogenic nanoparticles using peptide-polymer bioconjugates. Mol. Ther. **25**, 1655–1664 (2017). 28479234 10.1016/j.ymthe.2017.04.015 PMC5498834

[25] J. Chataway , Effects of ATX-MS-1467 immunotherapy over 16 weeks in relapsing multiple sclerosis. Neurology **90**, e955–e962 (2018). 29467307 10.1212/WNL.0000000000005118

[26] C. Sie, T. Korn, M. Mitsdoerffer, Th17 cells in central nervous system autoimmunity. Exp. Neurol. **262**, 18–27 (2014). 24681001 10.1016/j.expneurol.2014.03.009

[27] L. V. Sinclair , Phosphatidylinositol-3-OH kinase and nutrient-sensing mTOR pathways control T lymphocyte trafficking. Nat. Immunol. **9**, 513–521 (2008). 18391955 10.1038/ni.1603 PMC2857321

[28] C. M. Carlson , Kruppel-like factor 2 regulates thymocyte and T-cell migration. Nature **442**, 299–302 (2006). 16855590 10.1038/nature04882

[29] K. Hirota , Fate mapping of IL-17-producing T cells in inflammatory responses. Nat. Immunol. **12**, 255–263 (2011). 21278737 10.1038/ni.1993 PMC3040235

[30] B. Pulendran, P. S. Arunachalam, D. T. O’Hagan, Emerging concepts in the science of vaccine adjuvants. Nat. Rev. Drug Discov. **20**, 454–475 (2021). 33824489 10.1038/s41573-021-00163-y PMC8023785

[31] E. A. Gosselin, M. Noshin, S. K. Black, C. M. Jewell, Impact of excipients on stability of polymer microparticles for autoimmune therapy. Front Bioeng. Biotechnol. **8**, 609577 (2020). 33644005 10.3389/fbioe.2020.609577 PMC7906284

[32] G. W. Neff , Sirolimus-associated hepatotoxicity in liver transplantation. Ann. Pharmacother. **38**, 1593–1596 (2004). 15328399 10.1345/aph.1E165

[33] S. Senniappan , Sirolimus therapy in infants with severe hyperinsulinemic hypoglycemia. N. Engl. J. Med. **370**, 1131–1137 (2014). 24645945 10.1056/NEJMoa1310967

[34] B. Haliloglu , Sirolimus-induced hepatitis in two patients with hyperinsulinemic hypoglycemia. J. Clin. Res. Pediatr. Endocrinol. **10**, 279–283 (2018). 29217498 10.4274/jcrpe.5335 PMC6083472

[35] F. Wimmers , Monitoring of dynamic changes in Keyhole Limpet Hemocyanin (KLH)-specific B cells in KLH-vaccinated cancer patients. Sci. Rep. **7**, 43486 (2017). 28344338 10.1038/srep43486 PMC5361210

[36] B. Bielekova , Encephalitogenic potential of the myelin basic protein peptide (amino acids 83–99) in multiple sclerosis: Results of a phase II clinical trial with an altered peptide ligand. Nat. Med. **6**, 1167–1175 (2000). 11017150 10.1038/80516

[37] G. M. Delgoffe , The mTOR kinase differentially regulates effector and regulatory T cell lineage commitment. Immunity **30**, 832–844 (2009). 19538929 10.1016/j.immuni.2009.04.014 PMC2768135

[38] M. Battaglia, A. Stabilini, M. G. Roncarolo, Rapamycin selectively expands CD4+CD25+FoxP3+ regulatory T cells. Blood **105**, 4743–4748 (2005). 15746082 10.1182/blood-2004-10-3932

[39] M. Battaglia , Rapamycin promotes expansion of functional CD4+CD25+FOXP3+ regulatory T cells of both healthy subjects and type 1 diabetic patients. J. Immunol. **177**, 8338–8347 (2006). 17142730 10.4049/jimmunol.177.12.8338

[40] H. Chi, Regulation and function of mTOR signalling in T cell fate decisions. Nat. Rev. Immunol. **12**, 325–340 (2012). 22517423 10.1038/nri3198 PMC3417069

[41] M. A. Ackun-Farmmer, C. M. Jewell, Delivery route considerations for designing antigen-specific biomaterial strategies to combat autoimmunity. Adv. Nanobiomed. Res. **3**, 2200135 (2023). 36938103 10.1002/anbr.202200135 PMC10019031

[42] Y. Rui, H. B. Eppler, A. A. Yanes, C. M. Jewell, Tissue-targeted drug delivery strategies to promote antigen-specific immune tolerance. Adv. Healthc. Mater. **12**, e2202238 (2023). 36417578 10.1002/adhm.202202238 PMC9992113

[43] C. Krienke , A noninflammatory mRNA vaccine for treatment of experimental autoimmune encephalomyelitis. Science **371**, 145–153 (2021). 33414215 10.1126/science.aay3638

[44] S. S. Duffy, B. A. Keating, G. Moalem-Taylor, Adoptive transfer of regulatory T cells as a promising immunotherapy for the treatment of multiple sclerosis. Front Neurosci. **13**, 1107 (2019). 31680840 10.3389/fnins.2019.01107 PMC6803619

[45] I. Zubizarreta , Immune tolerance in multiple sclerosis and neuromyelitis optica with peptide-loaded tolerogenic dendritic cells in a phase 1b trial. Proc. Natl. Acad. Sci. U.S.A. **116**, 8463–8470 (2019). 30962374 10.1073/pnas.1820039116 PMC6486735

[46] J. Chataway , Effects of ATX-MS-1467 immunotherapy over 16 weeks in relapsing multiple sclerosis. Neurology **90**, e955–e962 (2018). 29467307 10.1212/WNL.0000000000005118

[47] L. Kappos , Induction of a non-encephalitogenic type 2 T helper-cell autoimmune response in multiple sclerosis after administration of an altered peptide ligand in a placebo-controlled, randomized phase II trial. The altered peptide ligand in relapsing MS study group. Nat. Med. **6**, 1176–1182 (2000). 11017151 10.1038/80525

[48] M. S. Freedman , A phase III study evaluating the efficacy and safety of MBP8298 in secondary progressive MS. Neurology **77**, 1551–1560 (2011). 21975206 10.1212/WNL.0b013e318233b240

[49] J. M. Gammon , Engineering the lymph node environment promotes antigen-specific efficacy in type 1 diabetes and islet transplantation. Nat. Commun. **14**, 681 (2023). 36755035 10.1038/s41467-023-36225-5 PMC9908900

[50] S.R. Allie , The establishment of resident memory B cells in the lung requires local antigen encounter. Nat. Immunol. **20**, 97–108 (2019). 30510223 10.1038/s41590-018-0260-6 PMC6392030

[51] D. J. Irvine, A. Aung, M. Silva, Controlling timing and location in vaccines. Adv. Drug Deliv. Rev. **158**, 91–115 (2020). 32598970 10.1016/j.addr.2020.06.019 PMC7318960

[52] M. Kuraoka , Recall of B cell memory depends on relative locations of prime and boost immunization. Sci. Immunol. **7**, eabn5311 (2022). 35522723 10.1126/sciimmunol.abn5311 PMC9169233

[53] N. Schwab, H. Wiendl, Learning CNS immunopathology from therapeutic interventions. Sci. Transl. Med. **15**, eadg7863 (2023). 37939164 10.1126/scitranslmed.adg7863

[54] M. Matloubian , Lymphocyte egress from thymus and peripheral lymphoid organs is dependent on S1P receptor 1. Nature **427**, 355–360 (2004). 14737169 10.1038/nature02284

[55] T. H. Pham, T. Okada, M. Matloubian, C. G. Lo, J. G. Cyster, S1P1 receptor signaling overrides retention mediated by G alpha i-coupled receptors to promote T cell egress. Immunity **28**, 122–135 (2008). 18164221 10.1016/j.immuni.2007.11.017 PMC2691390

[56] S. Flory , How to hit the allergy target: A critical appraisal of intralymphatic immunotherapy with practical recommendations on ultrasound-guided injections. Allergy **79**, 2222–2234 (2024). 38712754 10.1111/all.16138

[57] G. Senti , Intralymphatic allergen administration renders specific immunotherapy faster and safer: A randomized controlled trial. Proc. Natl. Acad. Sci. U.S.A. **105**, 17908–17912 (2008). 19001265 10.1073/pnas.0803725105 PMC2582048

[58] A. Chabot , Intralymphatic immunotherapy (ILIT) with bee venom allergens: A clinical proof-of-concept study and the very first ILIT in humans. Front Allergy **3**, 832010 (2022). 35386649 10.3389/falgy.2022.832010 PMC8974761

[59] J. Ludvigsson, J. Wahlberg, R. Casas, Intralymphatic injection of autoantigen in type 1 diabetes. N. Engl. J. Med. **376**, 697–699 (2017). 28199812 10.1056/NEJMc1616343

[60] R. Casas , Intra-lymphatic administration of GAD-alum in type 1 diabetes: Long-term follow-up and effect of a late booster dose (the DIAGNODE Extension trial). Acta Diabetol. **59**, 687–696 (2022). 35098372 10.1007/s00592-022-01852-9 PMC8995247

[61] J. Hillert , Treatment switching and discontinuation over 20 years in the big multiple sclerosis data network. Front Neurol **12**, 647811 (2021). 33815259 10.3389/fneur.2021.647811 PMC8010264

[62] L. Klotz, J. Antel, T. Kuhlmann, Inflammation in multiple sclerosis: Consequences for remyelination and disease progression. Nat. Rev. Neurol. **19**, 305–320 (2023). 37059811 10.1038/s41582-023-00801-6

[63] Assessment of ANK-700 in patients with relapsing remitting multiple sclerosis (MoveS-it). Clinicaltrials.gov Identifier: NCT04602390. https://clinicaltrials.gov/study/NCT04602390?tab=results. Accessed 15 August 2024.

[64] A. Walczak, M. Siger, A. Ciach, M. Szczepanik, K. Selmaj, Transdermal application of myelin peptides in multiple sclerosis treatment. JAMA Neurol. **70**, 1105–1109 (2013). 23817921 10.1001/jamaneurol.2013.3022

[65] R. Planas , GDP-l-fucose synthase is a CD4(+) T cell-specific autoantigen in DRB3*02:02 patients with multiple sclerosis. Sci. Transl. Med. **10**, eaat4301 (2018). 30305453 10.1126/scitranslmed.aat4301

[66] I. Jelcic , Memory B cells activate brain-homing, autoreactive CD4(+) T cells in multiple sclerosis. Cell **175**, 85–100.e123 (2018). 30173916 10.1016/j.cell.2018.08.011 PMC6191934

[67] M. T. Cencioni, M. Mattoscio, R. Magliozzi, A. Bar-Or, P. A. Muraro, B cells in multiple sclerosis—From targeted depletion to immune reconstitution therapies. Nat. Rev. Neurol. **17**, 399–414 (2021). 34075251 10.1038/s41582-021-00498-5

[68] J. J. Sabatino Jr., A. K. Probstel, S. S. Zamvil, B cells in autoimmune and neurodegenerative central nervous system diseases. Nat. Rev. Neurosci. **20**, 728–745 (2019). 31712781 10.1038/s41583-019-0233-2

