# Supplementary material for: Local control of T cell fate in lymph nodes safely and durably reverses myelin-driven autoimmunity
Source: Proc Natl Acad Sci U S A. 2025 Nov 3;122(45):e2409563122. doi: 10.1073/pnas.2409563122 (PMC12625849; doi:10.1073/pnas.2409563122)
Supplement: Supplementary file 1 — Appendix 01 (PDF) [file pnas.2409563122.sapp.pdf]

PNAS

Supporting Information for

Local control of T cell fate in lymph nodes safely and durably reverses myelin-driven autoimmunity

†Senta M. Kapnick[1,2], †Emily A. Gosselin[3], †Shannon J. Tsai[3], Robert S. Oakes[1,2], Zahra A. Habibabady[4,5], Marian A. Ackun-Farmmer[1,3], Sean T. Carey[1,3], Shrey A. Shah[1,3], Ruochen Shen[1,3], Eugene Froimchuk[3], Haleigh B. Eppler[3], Christopher J. Bridgeman[1,3], Alexis A. Yanes[3], Ryan A. McIlvaine[1,3], Maeesha Noshin[3], Lisa H. Tostanoski[3], Sheneil K. Black[3], Xiangbin Zeng[3], Agnes Azimzadeh[4,5], Richard N. Pierson III[4,5], Jonathan S. Bromberg[5,6,7], *Christopher M. Jewell[1,2,7,8]

†These authors contributed equally to this work

Christopher M. Jewell
Email: cmjewell@umd.edu

This PDF file includes:

Extended Materials and Methods
Figures S1 to S14
Tables S1 to S3
SI References

Extended Materials and Methods

Microparticle Synthesis and Characterization

PLGA MPs were assembled using double-emulsion/solvent evaporation, as previously described (1). Briefly, 0.75mg ($PLP_{139\text{-}151}$) or 1mg ($MOG_{35\text{-}55}$, $OVA_{323\text{–}339}$) of peptide was loaded into the inner aqueous phase in 500uL of water and sonicated for 30s at 12W with the organic phase comprised of 80mg PLGA in 5mL dichloromethane. 2mg of Rapamycin was included in the organic phase during sonication. For all formulations, the primary emulsion was homogenized with 40mL 2% PVA for 3min at 16,000RPM to form stabilized double emulsions. MPs were filtered through 40uM cell strainers and collected by centrifugation (5min, 5000xg, 4C), followed by three washes in MilliQ water. Final MP resuspension was performed in sterile PBS. For non-human primate studies, PLGA MPs were synthesized with 1mg ovalbumin (OVA) or keyhole limpet hemocyanin (KLH), and 2 mg Rapamycin following the procedure described above.

To measure loading of peptide and Rapa, a known volume of MPs was air-dried and dissolved in dimethyl sulfoxide (DMSO). Rapa loading was measured at 280nm by UV/Vis spectrophotometry using soluble Rapa for generation of standard curve. Peptide loading was measured using the micro bicinchoninic acid (mBCA) assay kit (Thermo Fisher Scientific) according to manufacturer’s instructions. Known peptide concentrations in solution were used to generate standard curves. Particle size was measured using an LA-950 laser diffraction size analyzer (Horiba Instruments).

Lyophilization and Reconstitution of Microparticles

MPs were lyophilized and resuspended as previously described (1). Briefly, MPs were resuspended at desired concentrations in cryoprotectant (1% trehalose in MilliQ water), aliquoted into glass vials, and flash frozen on dry ice. Samples were loaded into a FreeZone 6 L Console -84C Freeze Dryer (Labconco) and lyophilized for up to 24hrs. Vials were sealed and stored at room temperature, protected from light for over 90 days before reconstitution in sterile PBS.

Mice

6–12-week-old, female age-matched mice were used in all studies. C57BL/6J, Thy1.1 (B6.PL-Thy1a/CyJ), OT-II (Tg[TcraTcrb]425Cbn), 2D2 (Tg[Tcra2D2,Tcrb2D2]), and Foxp3/GFP (B6.Cg-Foxp3^{tm2Tch}/J) mice were purchased from The Jackson Laboratory. 2D2 Foxp3/GFP, and 2D2 Thy1.1 congenic mice were bred in University of Maryland facilities. All mice were housed in individually ventilated cages under specific pathogen-free conditions. Animal care and experiments were carried out using protocols approved and overseen by the University of Maryland Institutional Animal Care and Use Committee (IACUC) in compliance with state and federal guidelines and per the Association for Assessment and Accreditation of Laboratory Animal Care (AAALAC) expectations for animal care and use/ethics.

Intra Lymph Node (i.ln.) Microparticle Injections

Tolerogenic MP treatments were delivered directly to inguinal LNs using a non-surgical, injection technique as previously described (2). Briefly, 3-24hrs prior to injection, mice were shaved and remaining fur above the injection site was removed using a mild depilatory cream. A tracer dye (Evans blue) (Alfa Aesar) was injected subcutaneously on each side of the tail base. Mice received MPs in sterile PBS via 10uL injections containing 1mg of indicated MP formulations. For *i.ln.* treatments administered after disease onset (peak disease or chronic/late disease treatment), mice were assigned to normalized groups based on clinical score and weight loss to ensure similar disease status among *i.ln.* treatment groups.

RR-EAE and EAE Induction and Monitoring

RR-EAE was induced in 9-week-old female SJL/J mice (Jackson Laboratory) using emulsion containing Complete Freund’s Adjuvant (CFA) and [Ser^{140}]-$PLP_{139\text{-}151}$ (Hooke Laboratories) (3). Briefly, mice were anesthetized under isoflurane and injected at four sites in the subcutaneous space along the midline, totaling 200uL of emulsion per mouse.

EAE was induced in 10-week-old female C57BL/6J mice (Jackson Laboratory), as previously described (4). Briefly, 4mg/mL heat-inactivated tuberculosis (hiTB, nonviable desiccated Mycobacterium tuberculosis H37 Ra) was

combined with Incomplete Freund's Adjuvant (IFA) to produce Complete Freund's Adjuvant (CFA). 2mg/mL peptide ($MOG_{35–55}$) was prepared in sterile PBS. Solutions were mixed at a 50:50 ratio (v/v) and emulsified for 35sec using a probe sonicator at 87% amplitude (Qsonica, CL-18 1/8 in. probe). For EAE induction, mice were anesthetized under isoflurane and injected at two sites in the subcutaneous space along the midline, totaling 200uL of emulsion per mouse. EAE mice received 60ng of pertussis toxin (List Biological Laboratories) in sterile PBS *i.p.* two and 24hrs after emulsions.

RR-EAE and EAE mice were monitored daily for weight and paralysis, which was assessed using a clinical score rubric: 0, no symptoms; 0.5, partial tail paralysis; 1, paralyzed (limp) tail; 1.5, decrease in hind limb stability; 2, hind limb weakness and altered gait; 2.5, partial hind limb paralysis; 3, hind limb paralysis; 3.5, hind limb paralysis and trunk weakness; 4, hind limb paralysis and partial front limb paralysis; 4.5, hind and front limb paralysis; and 5, moribund. Humane end points include a score of 4 for two consecutive days or ≥ 4.5. Water (hydrogel) and food were placed on the cage floor level to accommodate paralysis during disease course. Disease incidence (symptomatic) was defined as the first day a mouse exhibited a clinical score >0; paralysis incidence was defined as the first day a mouse exhibited a clinical score ≥3; relapse incidence in RR-EAE was defined as the first day a mouse exhibited +1 increase in clinical score during the second or third wave of disease.

Ovalbumin Vaccination and Blood Collection

Mice were injected at indicated time points in the dorsal subcutaneous space with a total of 50uL sterile PBS containing whole ovalbumin protein (OVA, 250ug) (Worthington Biochemical Corporation) and CpG (50ug) (IDT). Blood was collected weekly via the facial vein into either MiniCollect CAT serum separator tubes or K_2EDTA collection tubes (Greiner Bio-One) for analyses of anti OVA IgG in serum or tetramer+ CD8 T cells in peripheral blood, respectively. Blood in serum separator tubes was allowed to fully clot, followed by centrifugation at 10,000xg at room temperature for 10 minutes. Serum was transferred to microcentrifuge tubes and stored at -80C until further use. Blood was processed for immediate use.

Adoptive Transfers

CD4 T cells from spleens and lymph nodes of donor mice were isolated using magnetic bead-based magnetic negative selection (StemCell) per manufacturer's instructions, or obtained via sorting on a BD FACS Aria II (BD Biosciences). 24hrs after *i.ln.* MP treatment, CD4 cells were resuspended in sterile PBS and adoptively transferred in 200uL into recipients by *i.v.* tail vein injection, totaling 3-5x10^6 donors/recipient mouse. Prior to adoptive transfer and where indicated, isolated T cells were labeled with 1µM carboxyfluorescein succinimidyl ester (CFSE, Invitrogen) or CellTrace Violet (CTV, Thermo Fisher Scientific) per manufacturer's instructions.

Tissue Processing

At the indicated time points, tissues were collected from mice induced with RR-EAE or EAE, mechanically dissociated through a 70um cell strainer, and centrifuged (5min, 500xg, 4C). Splenocytes were resuspended in ACK lysis buffer (Invitrogen) for erythrocyte lysis and washed with PBS for downstream applications. Cells were collected from spinal cords using hydraulic extrusion. Briefly, the spinal column was removed by cutting along the spine with a pair of scissors and straightened by placing an index finger on the proximal bend of the column. The tip of an 18G needle loaded onto a 10mL syringe filled with PBS was inserted into the proximal end of the spinal cavity. Steady pressure was applied to extrude the spinal cord. Spinal cords were further processed by dissociation through a 70um cell strainer and using Debris Removal Kit (Miltenyi) per manufacturer's instructions.

Ex vivo Restimulation and Cytokine Secretion Analysis

To assess antigen-specific responses by T cells, cells were collected from the inguinal (treated) and pooled axillary/brachial LNs mice. One million total cells collected from LNs were restimulated in completed RPMI 1640 (RPMI-1640 + 10% fetal bovine serum + 2mM L-glutamine + 10mM HEPES + 55μM β-mercaptoethanol + 100U Pen/Strep) supplemented with either MOG_{35-55} or $OVA_{323-339}$ peptides at 25μg/mL. Cells were cultured for 72 hours, after which supernatants were collected and stored at -80C for further analysis by ELISA.

Dendritic Cell:T Cell Co Culture

For dendritic cell (DC) isolation, spleens from C57BL/6J mice were digested using Spleen Dissociation Medium (StemCell) followed by magnetic bead-based CD11c positive selection (Miltenyi), both per manufacturer

instructions. 100x10^3 DCs were plated in 96 well plates in complete RPMI 1640 and treated with 100ng/mL lipopolysaccharide (LPS) (Millipore Sigma) and 10ug/well of MPs or 25ug/mL soluble MOG_{35-55}, where indicated. 24hrs after plating, CD4 T cells were isolated from spleens and LNs of 2D2 mice using magnetic bead-based negative selection (StemCell), per the manufacturer's instructions. 300x10^3 T cells/well were co-cultured with DCs and surface markers or proliferation were evaluated using flow cytometry three days after start of co-culture.

Enzyme-linked Immunosorbent Assay (ELISA)

IFNγ ELISAs (BD Biosciences) were performed according to the manufacturer's instructions. To calculate circulating anti OVA IgG concentrations in serum, indirect ELISAs were performed. 200ug/mL ovalbumin protein in PBS was coated onto high-binding microwell plates by incubating overnight at 4C. Coating solution was removed with wash buffer (PBS). Plates were blocked for 2hrs at room temperature in blocking solution (5% bovine serum albumin (BSA) in PBS). 1:10 serial dilutions of anti OVA IgG (R&D Systems) standard beginning at 1ug/mL in blocking solution were used. Serum samples were diluted 1:100 in blocking solution for the top dilution, followed by 1:10 serial dilutions per sample. 100uL of standards, blank, and samples were added to wells. Plates were sealed and incubated 2hrs at room temperature, followed by washing. 100uL of horseradish peroxidase (HRP)-conjugated IgG H+L (AbCam) in blocking solution was added to each well and incubated for one-hour at room temperature while rocking. IgG-HRP solution was with wash buffer. Plates were incubated with 50uL of prewarmed tetramethylbenzidine (TMB) for 15 minutes, after which 50uL of stop solution (2M H_2SO_4) was added to wells. Absorbance was read at 405nm using Tecan Spark microplate reader (Tecan Life Sciences). Anti OVA IgG concentrations were calculated by plotting absorbance versus known anti OVA IgG concentration to generate a standard curve.

Cell Staining and Flow Cytometry

For staining surface markers, cells were washed and blocked in FACS buffer (PBS + 1% FBS) in the presence of CD16/CD32. Viability (Live Dead NIR or aqua, Thermo Fisher) and surface marker staining (anti CD4, clone RM4-5; anti CD8a, clone 53-6.7; anti CD25, clone PC61; anti CD44, clone IM7; anti CD62L, clone MEL-14) were performed in FACS buffer at 4C for 20min, protected from light, followed by fixation with 2% paraformaldehyde. Intracellular staining (anti Foxp3, clone MF23; anti Rorγt, clone Q31-378; anti pS6, clone D57.2.2E) was performed using the Foxp3/Transcription Factor Staining Buffer Set (Thermo Fisher Scientific), per the manufacturer's instructions. Cell population frequencies are reported as percent of parent gates. Cell numbers within tissues are calculated based on number of cells within a defined gate recovered following acquisition/analyses of entire tissue. For detection of OVA-specific CD8 T cells following vaccination, blood collected from mice via facial vein was blocked and stained with a viability dye (LiveDead) (Thermo Fisher Scientific), anti CD8a, and SIINFEKL tetramer (PE, H-2Kb, NIH Tetramer Core) for 30 min at room temperature. ACK lysis to remove erythrocytes was performed before data acquisition. Data were acquired on either a FACS Celesta (BD Biosciences) or CytoFlex (Beckman Coulter). All data was analyzed using FlowJo software (BD Biosciences).

T_{reg} Suppression Assay

Naive effector T cells were obtained by sorting live, CD4+ CD44- CD62L+ CD25- T cells from spleens of healthy mice and stained using 1µM CellTrace Violet (CTV, Thermo Fisher Scientific) proliferation dye, per the manufacturer's instructions. 50x10^3 effector CD4 T cells were added to 96-well culture plates coated with 1ug/mL anti CD3 (clone 2C11) in the presence of 50x10^3 mitomycin C-treated (Millipore Sigma) antigen-presenting cells harvested via negative selection using anti FITC magnetic beads (Miltenyi) from spleens of healthy mice (5). T_{regs} were obtained by sorting CD4+ GFP+ T cells from Foxp3/GFP untreated mice or six days after MP treatment. Graded numbers of T_{regs} were sorted directly into wells seeded with effectors and APCs. Proliferation among effectors was quantified after three days of culture. All cell sorting was performed on a BD FACS Aria II using BD FACS Diva software (BD Biosciences).

Immunofluorescence Microscopy

To harvest spinal cords for microscopy, mice were anesthetized with 5% isoflurane and transcardially perfused (0.72 mL/min, 10 min per perfusate) using a peristatic pump (Ismatec Reglo ICC Digital Pump) with ice-chilled PBS supplemented with 10U/mL heparin (Alfa Aesar) to exsanguinate, followed by 4% paraformaldehyde in PBS. Spinal cords were dissected and post-fixed in 4% paraformaldehyde for 24 hours at 4°C and sequentially equilibrated in 10% then 30% sucrose in PBS. Tissues were embedded in tissue sectioning compound (Tissue-Tek O.C.T., Sakura

Finetek) for 30um coronal plane sectioning using a cryostat (Leica Microsystems, stage -15°C, sample -10°C). Sections were stained using primary antibodies: myelin basic protein (MBP, Santa Cruz Biotechnology, clone F-6, 0.002 µg/µL), and neurofilament 200 (NF200, Sigma-Aldrich, polyclonal, 0.0097 µg/µL) followed by goat anti-rabbit IgG Alexa Fluor 594 (Invitrogen, 0.008 µg/µL) and goat anti-mouse IgG1 Alexa Fluor 488 (Invitrogen, 0.008 µg/µL) and DAPI counterstain. Briefly, free-floating tissue sections were processed as follows: 1) 2x wash with Tris Buffered Saline + 0.025% Triton (TBS/Triton) 2) TBS block for 30 min with 0.02ug/uL fragmented antibodies (AffiniPure Unconjugated Fab, Jackson ImmunoResearch), 3) 2x wash with TBS/Triton followed by 10% goat serum block for 2hrs, 4) immunolabeled with primary antibodies for 18 hours, 5) washed twice (TBS/Triton) over four hours, 6) immunolabeled with secondary antibodies for 18 hours, 7) washed with TBS supplemented with DAPI for 1 hour, 8) wash with TBS over 18 hours. To facilitate diffusion of reagents and prevent photobleaching, staining and washes were carried out on a rotary shaker. Sections were transferred to slides, mounted in Fluoromount-G (Southern Biotech) with a coverslip, and stored at 4°C.

Fluorescent images were captured in batch using an automated stage (Olympus, #IX-83 ZDC autofocus module, X-Cite #XYLIS, 10x #UPLXAPO10X, Hamamatsu ORCA Flash 4.0 sCMOS, Prior Scientific) and stitching with frame auto focus for each channel was used. For quantification, three sections from spinal cord depths for each mouse, and five fields-of-view from each section were examined (i.e., an average of 15 unique anatomical regions) to calculate the lesion/myelin ratio (% volume/volume) for each mouse. Lesion/myelin ratio was calculated for each section by a custom automated ImageJ (National Institutes of Health) script: 1) threshold for channels, 2) convert channel to mask, 3) analyze Particles module to calculate the area of lesion (DAPI, limit set to only calculate hypercellular regions and ignore single nuclei) or myelin tract (MBP), 4) multiply area by section thickness to calculate volume then lesion volume to myelin volume ratio for each field-of-view. Representative images were chosen to match the mean lesion/myelin ratio for each cohort.

Real-time Quantitative Polymerase Chain Reaction (RT-qPCR)

RNA was isolated using the Quick-RNA Microprep Kit (Zymo Research) from cells sorted directly from treated mice or DC:T co-cultures and lysed in RNA lysis buffer (Zymo Research), per the manufacturer's instructions including DNA degradation with DNase I. RNA was diluted to 20ng/µL in RT-qPCR grade water (Thermo Fisher Scientific). cDNA was reverse transcribed using the High-Capacity cDNA Reverse Transcription Kit (Thermo Fisher Scientific). The qPCR reaction mix was prepared using TaqMan Gene Expression Assay probes in TaqMan Gene Expression Master Mix (Thermo Fisher Scientific). TaqMan probes included: actin beta (Actb); 18s rRNA (18s); Kruppel-like factor 2 (Klf2); C-C Chemokine receptor 7 (Ccr7); Selectin L (Sell); and Sphingosine-1-phosphate receptor 1 (S1pr1). qPCR was performed in a MicroAmp Optical 384-well reaction plate with optical adhesive film on a QuantStudio 7 Flex Real-Time PCR System (Applied Biosystems, MA, USA). Samples were normalized to *Actb* and *18s* RNA, and data are expressed as relative to untreated using the $2^{-\Delta\Delta CT}$ method.

Liquid Chromatography-Mass Spectrometry (LC-MS)

Lyophilized MPs, MOG peptide, and Rapa standards were dissolved in a solution of 1:4 water:acetonitrile, to achieve final concentrations of ~0.01 mM. Samples were filtered through a 3kDa cartridge filter to separate excess PLGA polymer from cargo. Separation was performed on an Imtakt Cadenza C18 column (2.0 x 50 mm i.d., 3 µm) using an Agilent 1100 LC system at room temperature with a sample injection volume of 5 µL. The operating flow rate of 0.25 mL/min was used with the following gradient conditions comprising of mobile phase A (0.1% formic acid in water) and B (100% methanol): 0-10% B at 0-2 min, 10-75% B at 2-7 min, 75-100% B at 7-9 min, 100% at 9-12 min, 100-10% from 13-15 min. Data was monitored using a UV wavelength range from 190-400 nm and collected on the atmospheric pressure ionization time-of-flight mass spectrometer (AccuTOF; JEOL) equipped with an ESI ion source at a resolving power of 6000 (FWHM). The AccuTOF MS settings were as follows: needle voltage = 2300 V, desolvating chamber temperature = 250°C, orifice 1 temperature = 100°C, orifice 1 V= 60 V, orifice 2 = 10 V, ring V = 20 V. The mass range in the measurements was from 400 to 3000 Da and ESI positive mode.

Proton Nuclear Magnetic Resonance (^1H NMR)

Lyophilized MPs, MOG peptide, and Rapa standards were dissolved in 100% deuterated dimethyl sulfoxide (DMSO-d_6), to final concentrations of ~0.1-0.5 mM. Proton NMR data was recorded on a Bruker AV 600 MHz NMR equipped with a Cryo-TCI probe. All 1D NMR spectra were acquired at 25ºC using the zg30 pulse program. NMR was displayed using Bruker TopSpin v4.1.3 and converted to text files prior to plotting in GraphPad Prism. To deduce structure information, the expanded spectra were compared based on previously published NMR data (6).

Non-Human Primate Studies

All protocols were approved by the Institutional Animal Care and Use Committee at the University of Maryland School of Medicine and were conducted in compliance with the National Institutes of Health *Guidelines for the Care and Use of Laboratory Animals*. Both OVA and keyhole limpet hemocyanin (KLH) are prototypic T-helper-dependent immunogens (7). Under general anesthesia with isoflurane and propofol, cynomolgus macaque monkeys received 5mg of KLH/Rapa or OVA/Rapa MPs by direct injection of 75µL through a 31-gauge needle into four visible femoral/inguinal lymph nodes through an inguinal incision. The incision was closed in two layers of absorbable suture material and the wound painted with antibiotic ointment prior to recovery and return to the colony. Bupivacaine (0.5%) was injected sub-dermally after wound closure for analgesia. Peripheral blood was collected before study initiation and then weekly for nine weeks under chemical restraint/sedation with intramuscular ketamine. KLH/Rapa and OVA/Rapa MP-treated animals underwent challenge with KLH or OVA as an *i.m.* vaccine formulated in aluminum hydroxide (alum) 14 and 35 days after *i.ln.* treatment. Biochemical and hematologic measurements were performed on dedicated clinical veterinary assay platforms in the Azimzadeh/Pierson laboratory. Antibody responses to OVA and KLH were measured by ELISA. Reference control groups did not receive an *i.ln.* procedure but underwent serial weekly peripheral blood collection for hematologic and biochemical assays during immunization with KLH-alum, followed months later by OVA-alum immunization.

Quantification and Statistical Analysis

Statistical analyses were performed in Prism 10 (GraphPad Software) or JMP Statistical Software (SAS Institute). Figure legends specify the statistical tests used to determine P-values and "n" values, indicating the number of individual mice.

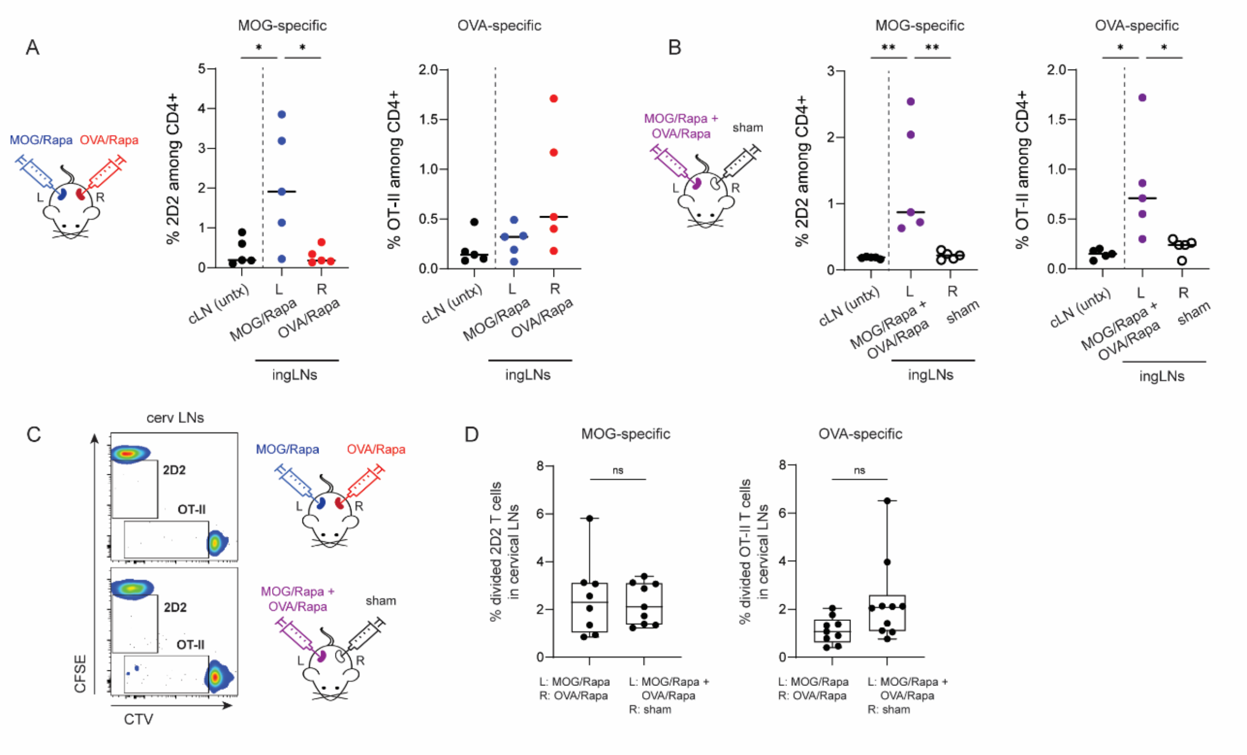

Figure S1. (A) Frequency of donor, antigen-specific 2D2 (left panel) or OT-II (right panel) T cells among total CD4+ in cervical (cLN) or inguinal LNs (ingLNs) treated with either MOG/Rapa (left LN, L) or OVA/Rapa (right LN, R) MPs, three days after transfer. **(B)** Frequency of donor, antigen-specific 2D2 (left panel) or OT-II (righ panel) T cells among total CD4+ in cervical (cLN) or inguinal LNs (ing LNs) treated with MOG/Rapa + OVA/Rapa MPs (left LN, L) or sham injections (right LN, R), three days after transfer. **(C)** Proliferative dye traces in cells harvested from cervical LNs for mice differentially treated in left (L) and right (R) LNs. **(D)** Frequency of divided cells among 2D2 (left panel) or OT-II T cells (right panel) in cervical LNs, three days after transfer. Single data points for cervical LNs represent pools from individual mice. Single data points for treated LNs represent individual LNs. n=4-8/treatment group. Error bars represent mean ± SD. ns=not significant, *p<0.05, **p<0.01, ***p<0.001 by One-way ANOVA Tukey−Kramer multiple comparisons test or Student's t-test.

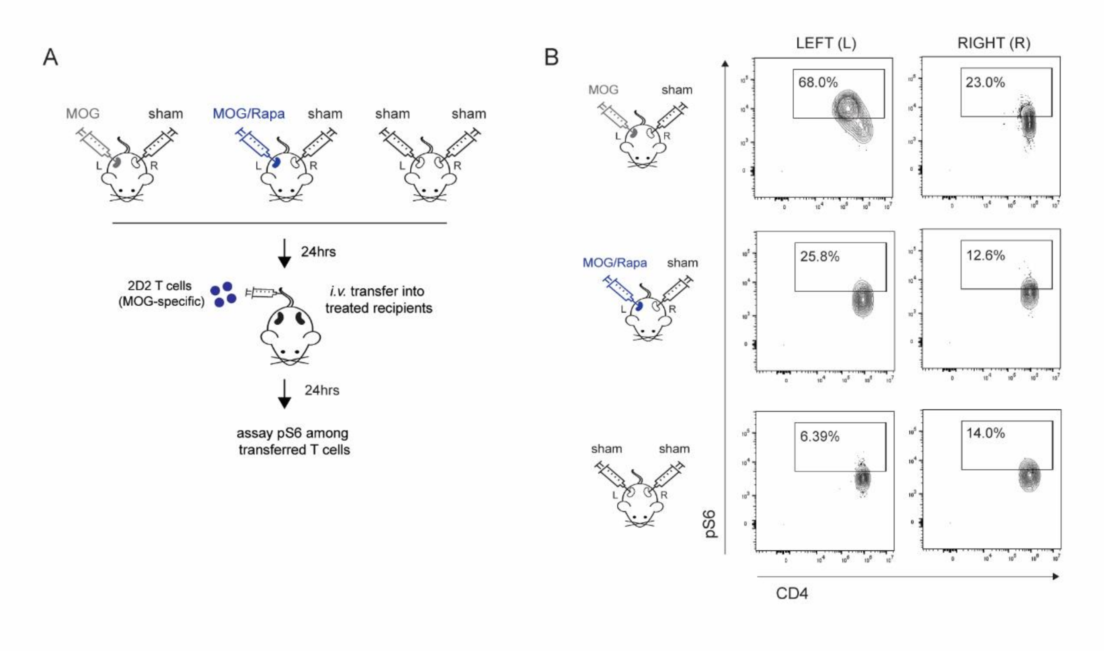

Figure S2. (A) Schematic of transfer studies and treatment groups for pS6 analysis. **(B)** Treatment cohorts and gating strategy for quantifying the frequency of pS6+ cells among 2D2 T cells recovered from left (L) or right (R) LNs.

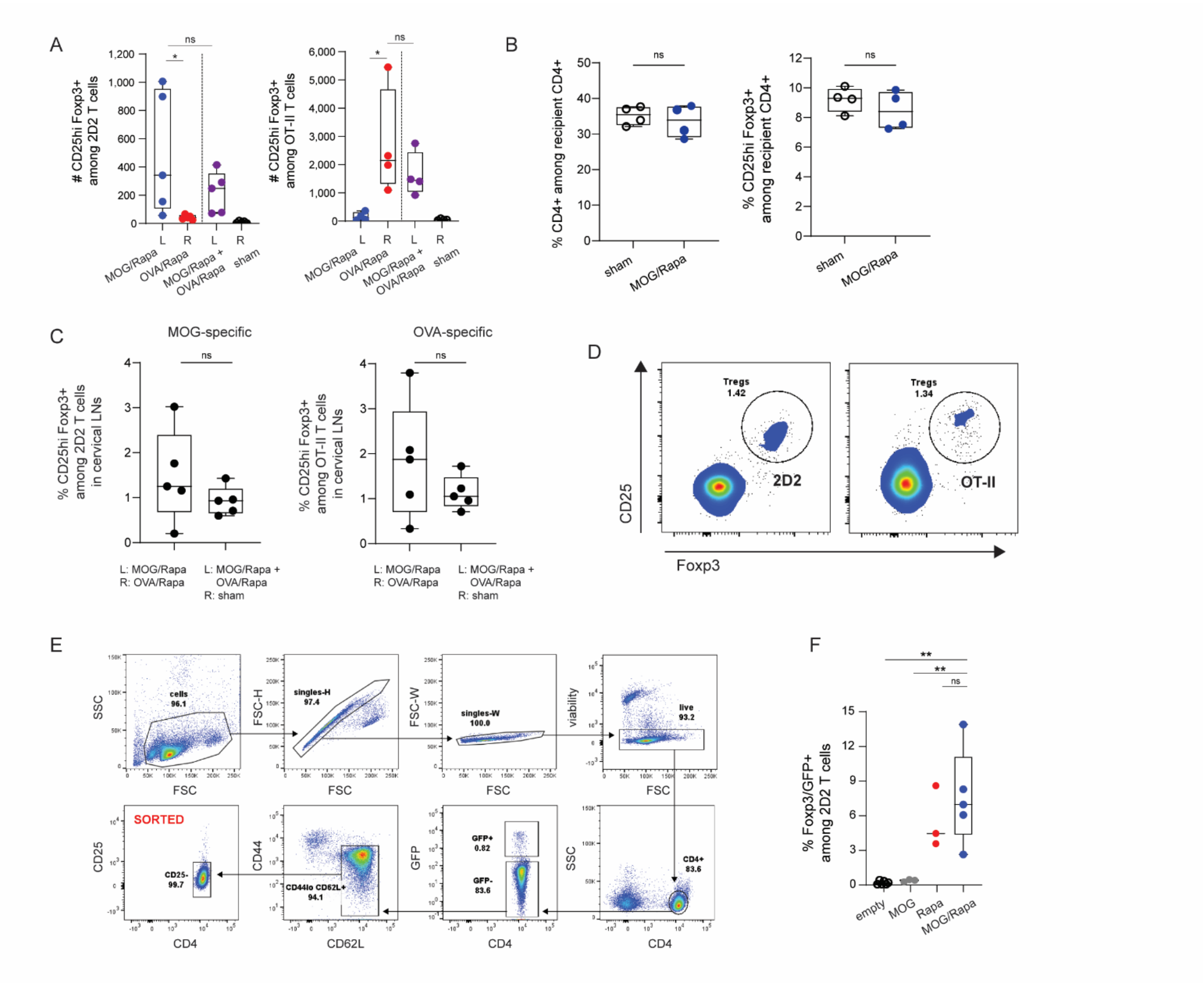

Figure S3. (A) Quantification of the number of T_{regs} among 2D2 (left panel) or OT-II CD4^{+} T cells (right panel) in treated LNs, six days after transfer. Single data points represent LNs. Dashed lines separate treatment strategies for individual mice. **(B)** Frequency of CD4+ (left panel) and T_{regs} (right panel) among endogenous cells in healthy, sham-injected or MOG/Rapa MP-treated recipient mice. **(C)** Frequency of T_{regs} among donor 2D2 (left panel) or OT-II (right panel) recovered from cervical (untreated) LNs, six days after transfer. Single data points for cervical LNs represent pools from individual mice. **(D)** Pre-existing T_{regs} among donor 2D2 (left panel) or OT-II T cells (right panel) in adoptive transfer studies. **(E)** Representative gating strategy for sort of T_{reg}-depleted, naive, CD4 T cells for *in vivo* studies. **(F)** Frequency of T_{regs} recovered among transferred 2D2 T cells in mice. n=4-8/treatment group. Error bars represent mean ± SD. ns=not significant, *p<0.05, **p<0.01, ***p<0.001 by One-way ANOVA Tukey−Kramer multiple comparisons test or Student's t-test.

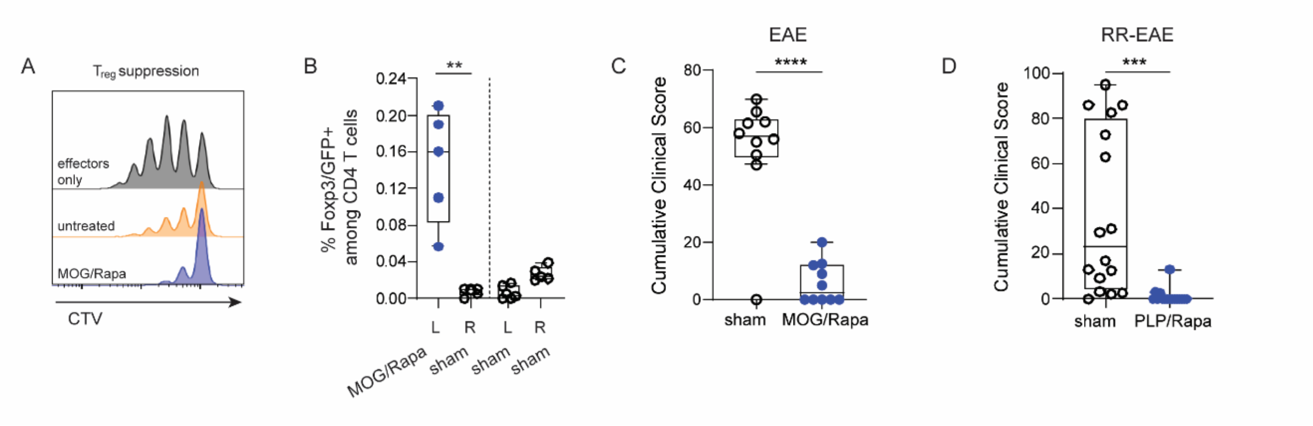

Figure S4. (A) Representative histogram illustrating proliferated effector CD4 T cells in T_{reg} suppression assays; effector:T_{reg} ratio = 1:0 (black), 1:1 (orange and blue). **(B)** Frequency of Foxp3/GFP+ T_{regs} among total CD4 T cells, 28 days after transfer. Single data points represent individual LNs. Dashed lines separate treatment schemes for individual mice, n=5/tx gp. Cumulative clinical score in mice treated with **(C)** sham and PLP/Rapa MPs or **(D)** sham and MOG/Rapa MPs on day -28 before EAE or RR-EAE induction over the 36- or 55-day monitoring periods, respectively. sham n=16, MOG/Rapa MPs n=8, PLP/Rapa MPs, n=15. Error bars represent mean ± SD. ns=not significant, **p<0.01, ***p<0.001, ****p<0.0001 by One-way ANOVA Tukey−Kramer multiple comparisons test, or Student's t test.

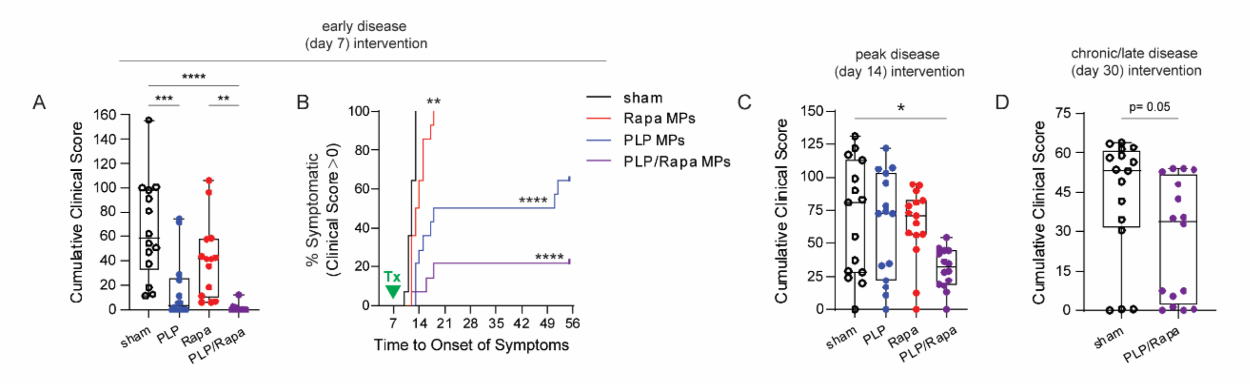

Figure S5. (A) Cumulative clinical score throughout the 55-day monitoring period in sham, PLP, Rapa, and PLP/Rapa mice treated day 7 post RR-EAE induction. **(B)** Time to symptomatic onset in mice treated day 7 (Tx). **(C)** Cumulative clinical score throughout the 55-day monitoring period in mice treated day 14 post RR-EAE induction. **(D)** Cumulative clinical score throughout the 55-day monitoring period in mice treated day 30 post RR-EAE induction. Error bars represent mean ± SD. *p<0.05, **p<0.01, ***p<0.001, ****p<0.0001 by one-way ANOVA with Tukey-Kramer post hoc test, or Wilcoxon test for Kaplan-Meier analysis.

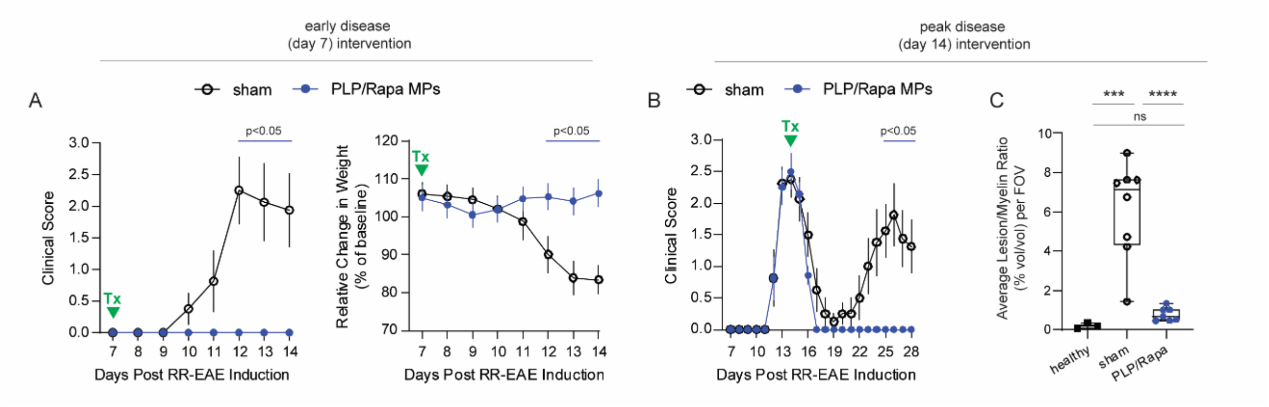

Figure S6. (A) Clinical score (left panel) and weight loss (right panel) in RR-EAE mice treated (Tx) on day 7 post RR-EAE induction. **(B)** Clinical score in RR-EAE mice treated (Tx) on day 14 post RR-EAE induction. **(C)** Average lesion/myelin ratio per FOV per mouse. Data for each intervention is representative of two or more similar experiments. Error bars for clinical score and weight loss represent mean ± SEM. Error bars in box and whiskey plots represent mean ± SD. ns=not significant, ***$p<0.001$, ****$p<0.0001$ determined using Steel-Dwass test (clinical scores), Two-way ANOVA with Tukey-Kramer post hoc test (weight loss), or One-way ANOVA Tukey−Kramer multiple comparisons test.

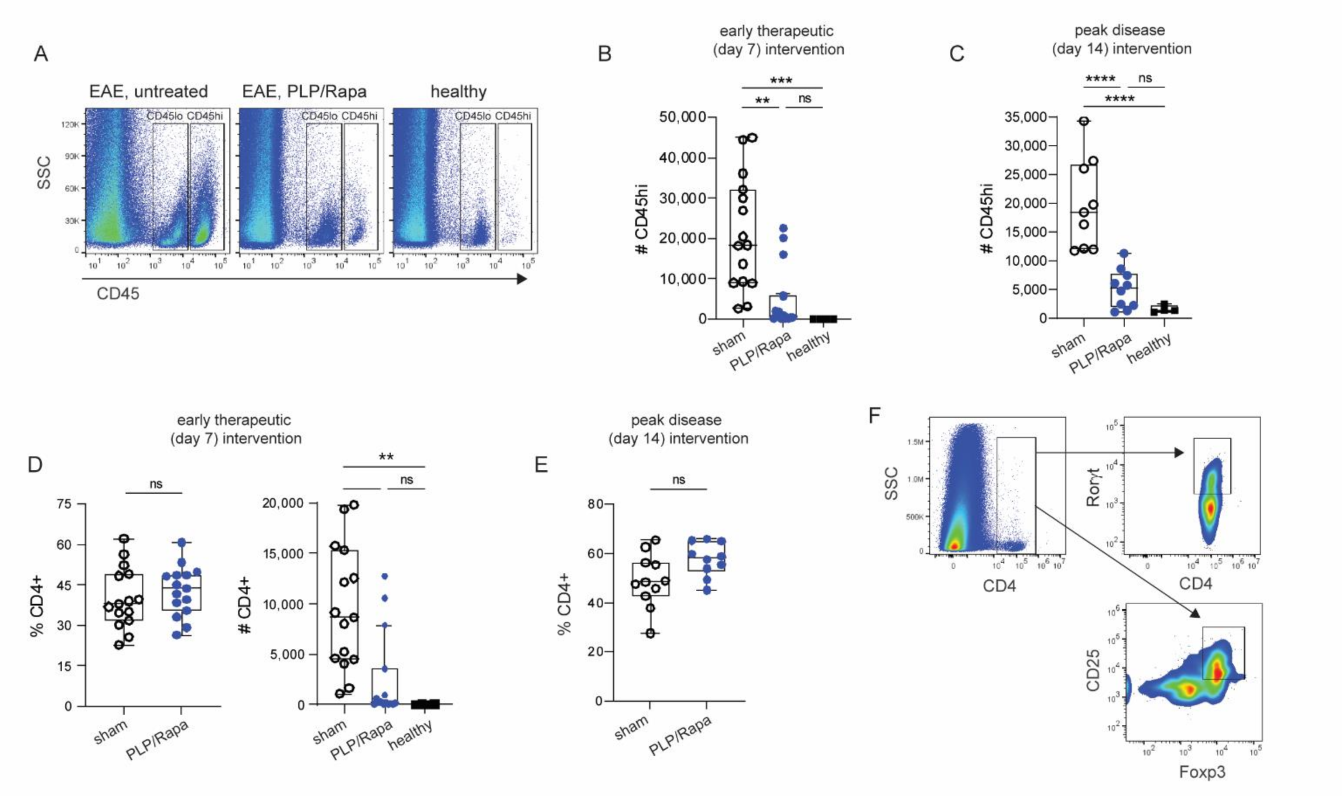

Figure S7. (A) Representative gating strategy for identification of infiltrating leukocytes (CD45hi) in spinal cords of mice. **(B)** Quantification of CD45hi cells in spinal cords of mice treated on day 7 post RR-EAE induction; tissue harvested on day 14. **(C)** Quantification of CD45hi cells in spinal cords of mice treated on day 14 post RR-EAE induction; tissue harvested on day 28. **(D)** Frequency (left panel) and number (right panel) of CD4 cells in spinal cords of mice treated on day 7 post RR-EAE induction; tissue harvested on day 14. **(E)** Frequency of CD4 T cells in spinal cords of mice treated on day 14 post RR-EAE induction; tissue harvested on day 28. **(F)** Representative gating strategy for identification of T_H17 and T_{regs} among CD4+ T cells isolated from spinal cords. Error bars in all panels represent mean ± SD. ns=not significant, **p<0.01, ***p<0.001, ****p<0.0001 by One-way ANOVA Tukey−Kramer multiple comparisons or Student's t-test.

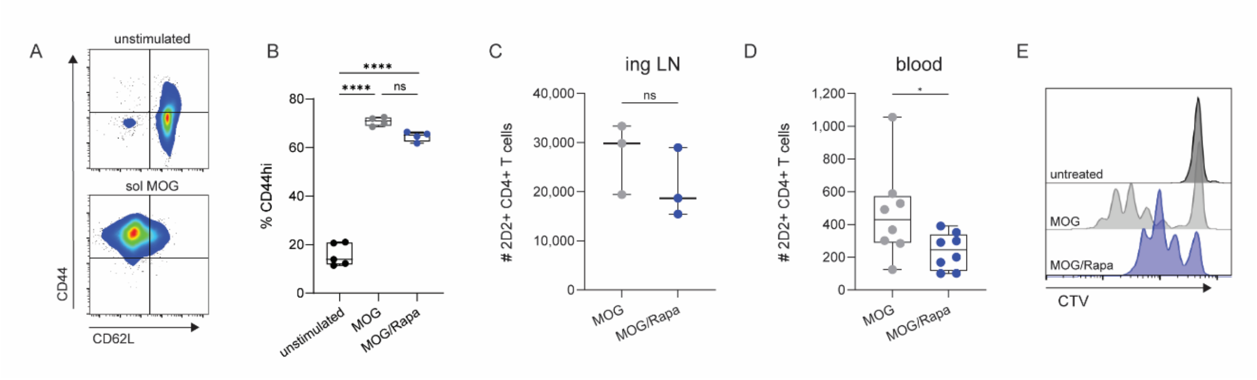

Figure S8. (A) Flow cytometric analyses of CD44 and CD62L surface marker expression. **(B)** Frequency of CD44hi CD4 T cells three days after start of DC:T cell *in vitro* co-culture assays. **(C)** Number of 2D2 T cells in treated, inguinal LNs six days post transfer into MP-treated recipients. **(D)** Number of 2D2 T cells in the blood six days post transfer into MP-treated recipients. Data are representative of two or more experiments. **(E)** Representative histograms illustrating proliferated 2D2 T cells in DC:T cell *in vitro* co-cultures, three days after start of assay. Error bars represent mean ± SD. ns=not significant, *$p<0.05$, ****$p<0.0001$ by One-way ANOVA Tukey−Kramer multiple comparisons or Student's t-test.

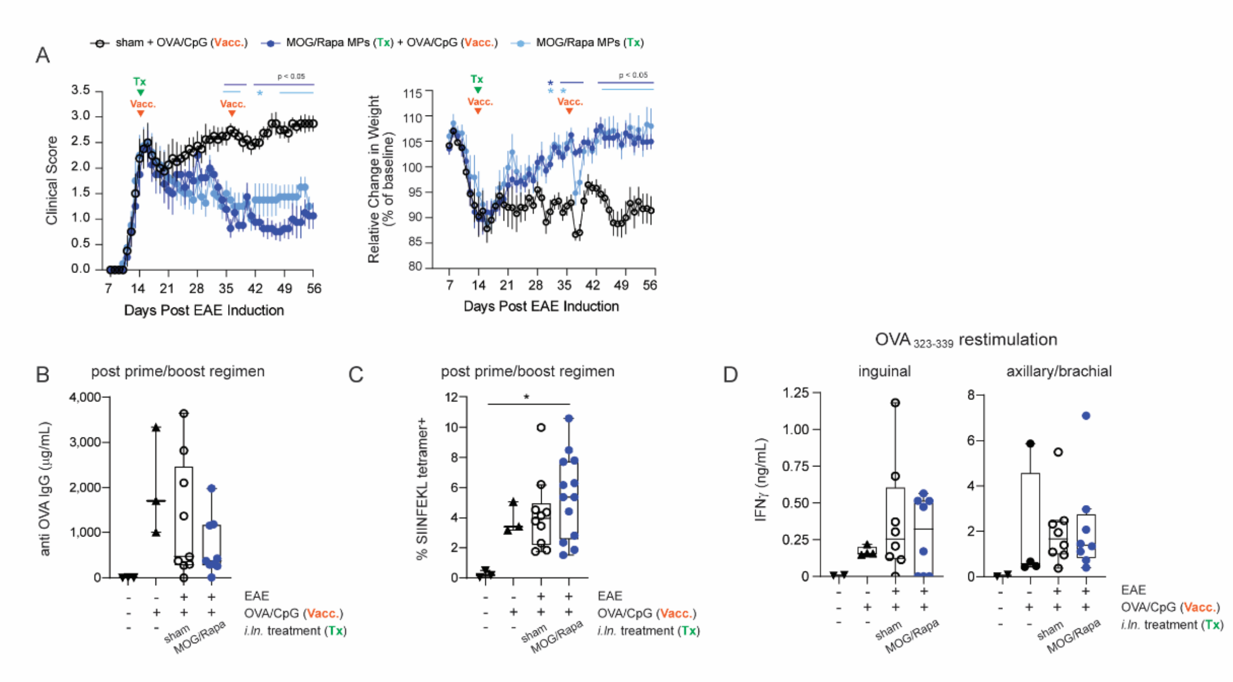

Figure S9. (A) Clinical score (left) and relative change in weight (right) following sham or MOG/Rapa *i.ln.* treated, EAE, OVA/CpG-vaccinated (dark blue) or treated, EAE, non-vaccinated mice (light blue). **(B)** Anti OVA IgG in serum following post *i.ln.* treatment and prime/boost vaccination course (day 28 post vaccination/day 43 post EAE induction). **(C)** Percent of SIINFEKL tetramer+ cells among CD8 T cells following *i.ln.* treatment and prime/boost vaccination course (day 28 post vaccination/day 43 post EAE induction). **(D)** IFNγ secretion by cells recovered from inguinal (left) or axillary/brachial (right) LNs following restimulation with $OVA_{323\text{-}339}$ peptide. Sham, n=9-10. MOG/Rapa MPs, n=9-13. Error bars for clinical score and relative weight loss represent mean ± SEM. Error bars in box and whisker plots represent mean ± SD. *p<0.05 by Steel-Dwass test (clinical scores), Two-way ANOVA with Tukey-Kramer post hoc test (weight loss), or One-way ANOVA Tukey−Kramer multiple comparisons test.

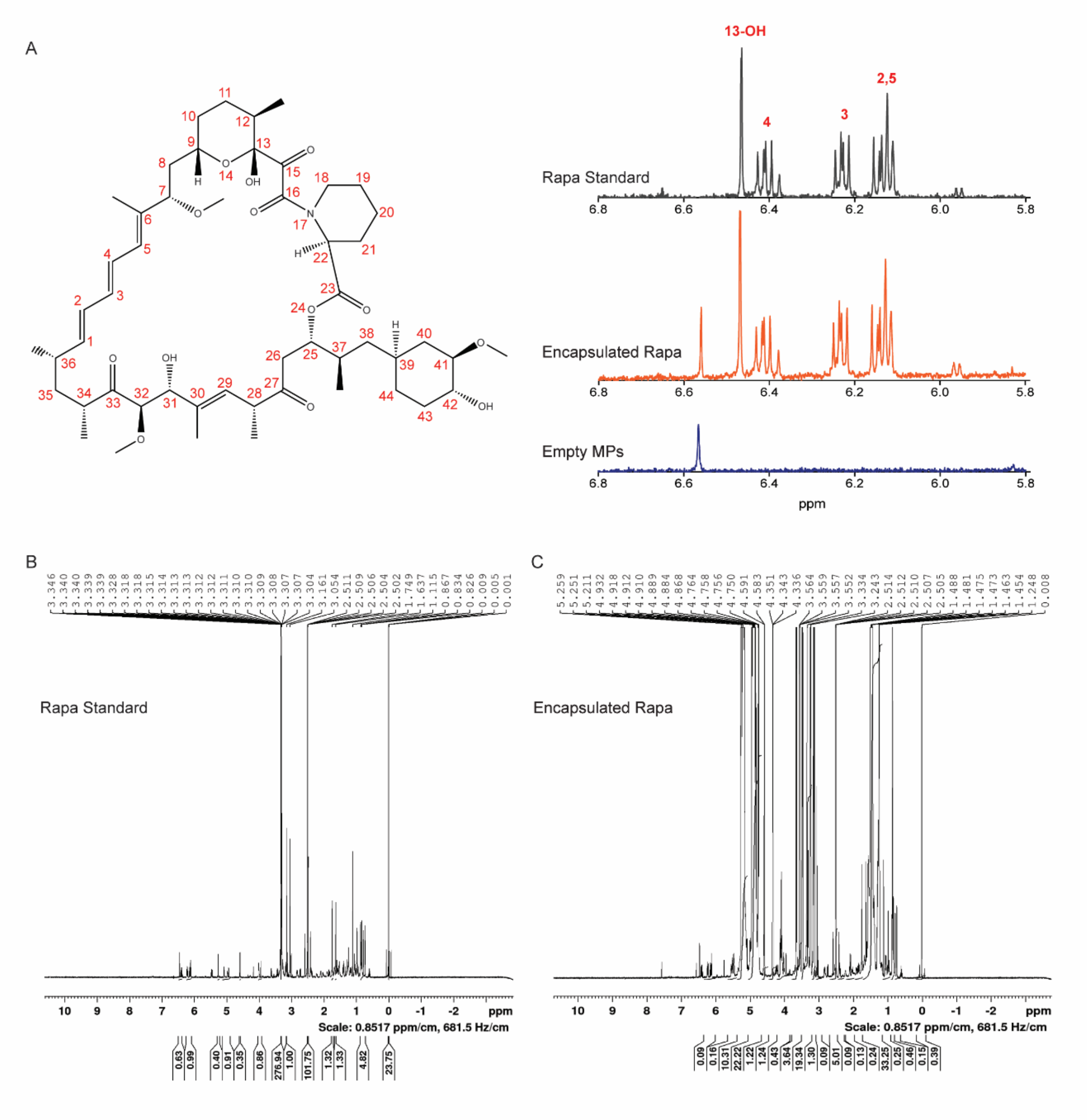

Figure S10. (A) Expanded ^{1}H-NMR spectra (600 MHz, DMSO-d6) for soluble Rapa standard (black), encapsulated Rapa recovered from MPs (orange – encapsulated Rapa), and empty MPs (blue). Relevant proton shifts are denoted in red in the Rapamycin structure. This region of the ^{1}H NMR spectra was selected to reduce noise from the MP signal. Full spectra of soluble Rapa standard **(B)** and encapsulated Rapa **(C)** recovered from MPs**.** The chemical shifts of the expanded regions of Rapa and Rapa MPs were identical, except for the appearance of a peak at 7.61 ppm, which correlates to an identical peak in the empty MP spectra. Data are representative of two or more experiments.

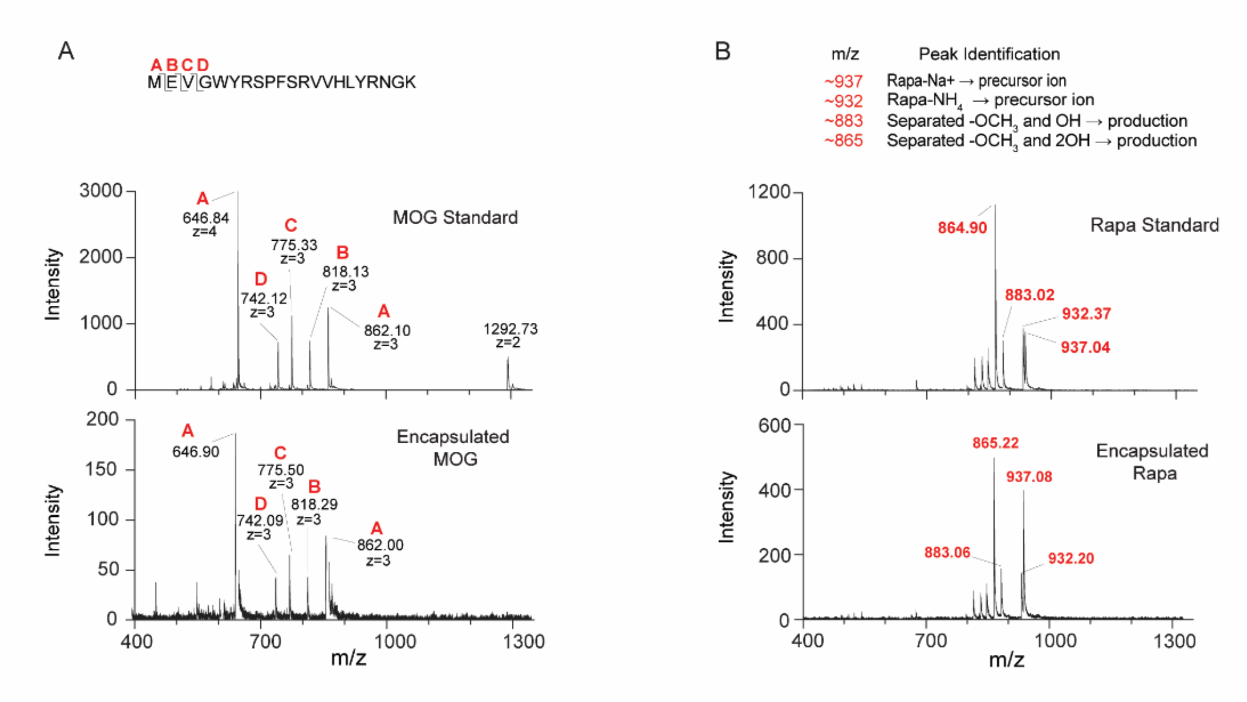

Figure S11. Mass spectrometry analysis of **(A)** soluble MOG standard (top panel) and encapsulated MOG recovered from MPs (bottom panel), where spectrum exhibits peaks corresponding to peptide fragmentation indicated by letters A-D. **(B)** Soluble Rapa standard (top panel) and encapsulated Rapa recovered from MPs (bottom panel), where spectrum exhibits peaks corresponding to fragmentation indicated in provided table.

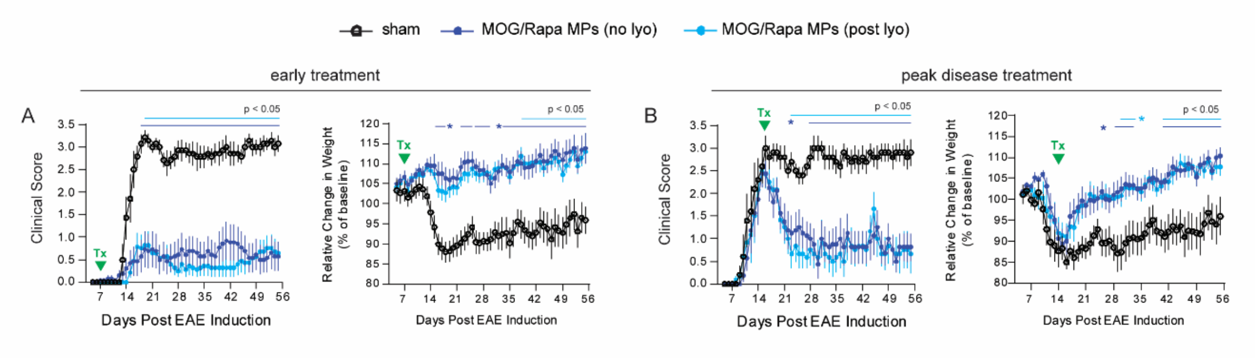

Figure S12. Clinical scores and weight loss in EAE mice following **(A)** early therapeutic or **(B)** peak disease treatment (Tx) regimens with freshly-prepared MOG/Rapa MPs (no lyo, dark blue), lyophilized, stored and reconstituted MOG/Rapa MPs (post lyo, light blue), or sham injections (black). Error bars for clinical score represent mean ± SEM. Sham, n=11. MOG/Rapa MPs, n=12-15 each. Data are representative of two experiments. *$p<0.05$ by Steel-Dwass test (clinical scores), or Two-way ANOVA with Tukey-Kramer post hoc test (weight loss).

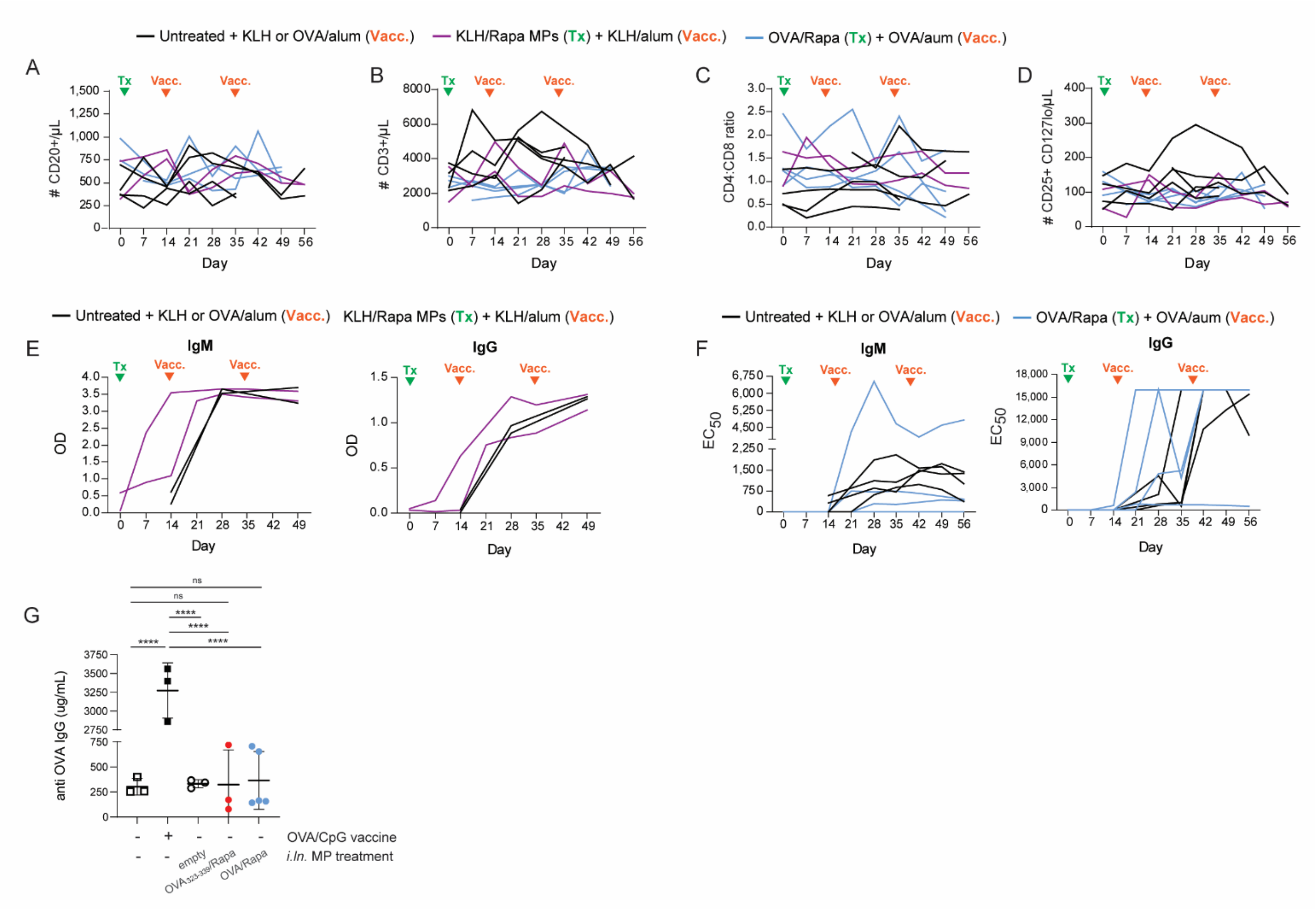

Figure S13. Quantification of circulating lymphocyte subsets/µL: **(A)** CD20+, **(B)** CD3+, **(C)** CD4:CD8 T cell ratio, **(D)** CD25+ CD127lo. **(E)** Anti KLH IgM (left panel) and IgG (right panel) in serum. **(F)** Anti OVA IgM (left panel) and IgG (right panel) in serum. KLH/Rapa MPs, n=2; OVA/Rapa MPs, n=4; untreated, n=6. **(G)** Anti OVA IgG in serum of healthy mice 35 days after vaccination or indicated MP treatments. n=3/gp. ns=not significant, ****p<0.0001 by One-way ANOVA Tukey−Kramer multiple comparisons test.

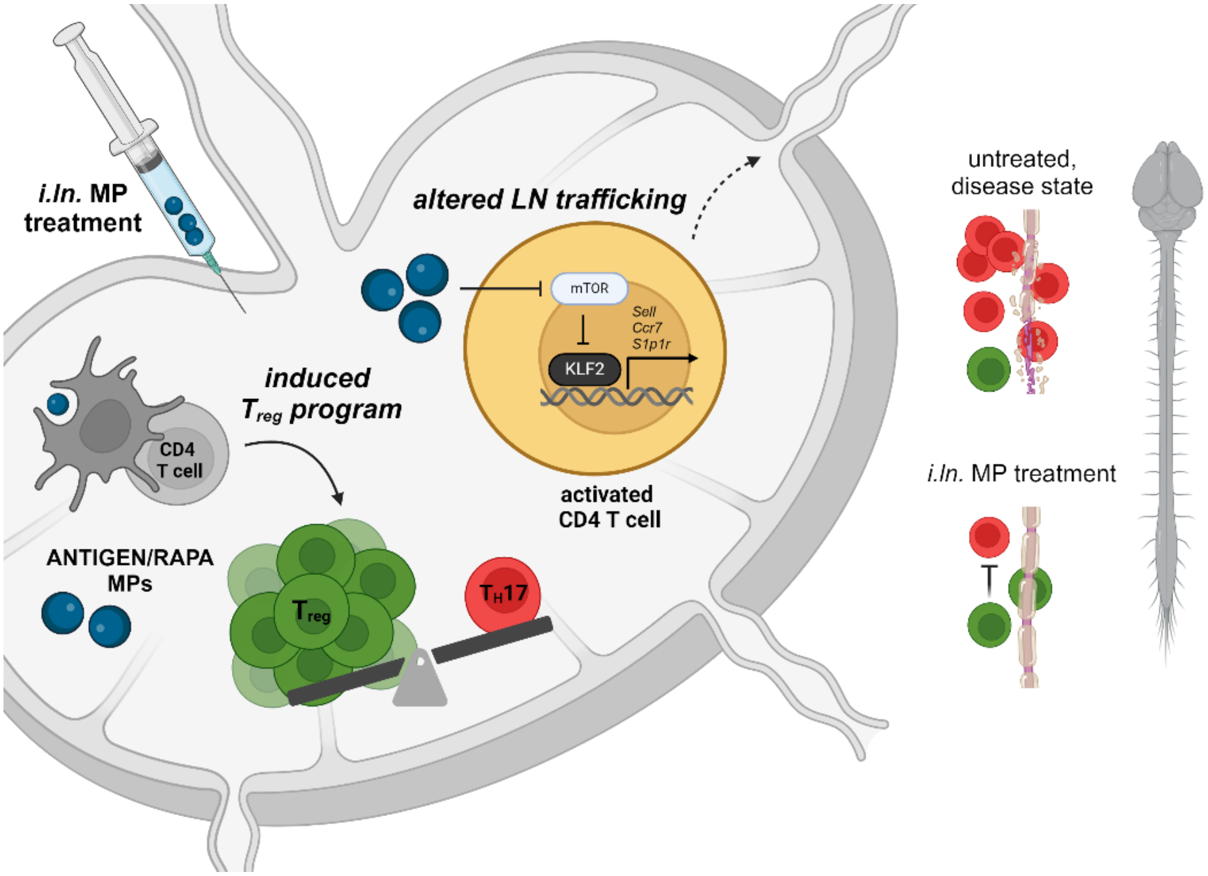

Figure S14. Direct lymph node injection exploits local dosing and MP retention to guide T cell fates and achieve durable efficacy with a single treatment in multiple preclinical models.

MP	Diameter (μm) ± SD	μg $PLP_{139-151}$ /mg MP ± SD	μg Rapa/mg MP ± SD
$PLP_{139-151}$	2.70 ± 0.56	4.08 ± 1.31	N/A
Rapa	2.93 ± 0.85	N/A	17.63 ± 5.36
$PLP_{139-151}$/Rapa	3.32 ± 0.84	3.89 ± 1.34	18.40 ± 6.33

Table S1. Properties of PLP, Rapa, and PLP/Rapa MPs utilized in studies. Measurements were pooled from three or more repeat particle synthesis and characterization experiments.

MP	Particle Prep	Diameter (µm) ± SD	µg MOG_{35-55} /mg MP ± SD	µg Rapa/mg MP ± SD
MOG_{35-55} /Rapa	no lyophilization	3.95 ± 1.47	9.43 ± 3.68	16.6 ± 3.18
MOG_{35-55}/Rapa	post-lyophilization	3.48 ± 1.24	10.70 ± 1.42	15.1 ± 4.80

Table S2. Properties of MOG/Rapa MPs pre- (no lyophilization) and post-lyophilization, storage, and reconstitution for use in *in vivo* studies. Measurements were pooled from three or more repeat particle synthesis and characterization experiments.

MP	Diameter (μm) ± SD	μg protein/mg MP ± SD	μg Rapa/mg MP ± SD
KLH /Rapa	3.90 ± 2.49	1.0	16.3
OVA /Rapa	3.75 ± 1.5	9.4 ± 0.4	16.1 ± 0.3

Table S3. Properties of KLH/Rapa and OVA/Rapa MPs for cynomolgus macaque safety study.

SI References

1. Gosselin EA, Noshin M, Black SK, & Jewell CM (2020) Impact of Excipients on Stability of Polymer Microparticles for Autoimmune Therapy. *Front Bioeng Biotechnol* 8:609577.
2. Andorko JI, Tostanoski LH, Solano E, Mukhamedova M, & Jewell CM (2014) Intra-lymph node injection of biodegradable polymer particles. *J Vis Exp* (83):e50984.
3. McRae BL*, et al.* (1992) Induction of active and adoptive relapsing experimental autoimmune encephalomyelitis (EAE) using an encephalitogenic epitope of proteolipid protein. *J Neuroimmunol* 38(3):229-240.
4. Robinson AP, Harp CT, Noronha A, & Miller SD (2014) The experimental autoimmune encephalomyelitis (EAE) model of MS: utility for understanding disease pathophysiology and treatment. *Handb Clin Neurol* 122:173-189.
5. Gomez-Rodriguez J*, et al.* (2014) Itk-mediated integration of T cell receptor and cytokine signaling regulates the balance between Th17 and regulatory T cells. *J Exp Med* 211(3):529-543.
6. Cao S, Zhou XB, Yang YS, Zhong W, & Sun TM (2014) Selective Substitution of 31/42-OH in Rapamycin Guided by an in Situ IR Technique. *Molecules* 19(6):7770-7784.
7. F W*, et al.* (2017) Monitoring of dynamic changes in Keyhole Limpet Hemocyanin (KLH)-specific B cells in KLH-vaccinated cancer patients. *Scientific reports* 7.
